# An improved group teaching optimization algorithm for global function optimization

**DOI:** 10.1038/s41598-022-15170-1

**Published:** 2022-07-04

**Authors:** Yanjiao Wang, Jieru Han, Ziming Teng

**Affiliations:** 1 School of Electrical Engineering, Northeast Electric Power University, Jilin, 132012 China; 2 College of Communication Engineering, Jilin University, Jilin, 130012 China

**Keywords:** Computer science, Information technology

## Abstract

This paper proposes an improved group teaching optimization algorithm (IGTOA) to improve the convergence speed and accuracy of the group teaching optimization algorithm. It assigns teachers independently for each individual, replacing the original way of sharing the same teacher, increasing the evolutionary direction and expanding the diversity of the population; it dynamically divides the students of the good group and the students of the average group to meet the different needs of convergence speed and population diversity in different evolutionary stages; in the student learning stage, the weak self-learning part is canceled, the mutual learning part is increased, and the population diversity is supplemented; for the average group students, a new sub-space search mode is proposed, and the teacher's teaching method is improved to reduce the diversity in the population evolution process. and propose a population reconstruction mechanism to expand the search range of the current population and ensure population diversity. Finally, the experimental results on the CEC2013 test suite show that IGTOA has clear advantages in convergence speed and accuracy over the other five excellent algorithms.

## Introduction

In many engineering areas, in order to obtain the maximum economic or social benefits, the best solution needs to be sought under certain conditions, such as: In the issue of cargo transport, the planned transport scheme meets both the quantity and loading conditions and minimizes total transport cost; In the process of product production, it is required to reduce the use of manpower and equipment to maximize the profit while meeting the product specifications. Mathematically, the essence of this process is the solution of optimization problems^1^. Due to the increasingly complex optimization problems proposed in the fields of science, engineering, and economics, even if complex mathematical models are built, it is difficult to obtain more desirable results. However, scholars have found that creatures in nature can accomplish complex tasks such as predation, risk avoidance, and evolution by assembling in groups, collaborating with each other, interacting, and other simple actions^2^. It presents a kind of group intelligence from which people take inspiration and design multiple evolutionary algorithms that can better solve optimization problems. Therefore, the swarm intelligence evolutionary algorithm has become the most effective and widely used method at present^3^.

At present, in engineering application fields, such as surface roughness modeling and optimization of tungsten-copper alloys in micro-milling processes^4^; a decision-making framework for dynamic scheduling of cyber-physical production systems based on digital twins^5^; evolving fuzzy models of shape memory alloy wire actuators^6^, the representative group intelligent evolution algorithm is mainly as follows: Genetic algorithm (GA), Differential Evolution (DE) algorithm , Particle Swarm Optimization (PSO) algorithm, Whale Optimization algorithm (WOA), Grey Wolf Optimizer (GWO), Artificial Bee Colony Algorithm (ABC) algorithm. Among them, the GA^7^ is a biological evolution process that simulates the natural selection and genetic mechanism of Darwin's theory of biological evolution, chromosomes are the main carrier of genetic material, that is, a collection of multiple genes, constitutes the genetic operation of genetic algorithm through selection, crossover and mutation. The DE^8^ is proposed based on the evolutionary idea of genetic algorithm, algorithm variant vectors are generated by the parent difference vector and crossed with the parent individual vector to generate new individual vectors, selected directly with its parent individual. The PSO algorithm^9^ is inspired by the social behavior of flocks or fish, each individual in a particle population is called a particle, representing a position or possible candidate solution in a multidimensional search space, updating positions by cooperating with each other until the optimal solution is found. The WOA^10^ is based on the behavior of whale prey, the location of each whale represents a viable solution, and during each generation of swimming, the whales randomly choose driving or rounding up to gradually approach their prey. The GWO^11^ is a group intelligent optimization algorithm for simulating its predator behavior, the algorithm assigns predation tasks such as encircling, chasing, and attacking gray wolves to different levels of gray wolves according to the gray wolf's social level to complete the predation behavior, thereby achieving global optimization. The ABC algorithm^12^ is inspired by the honey harvest behavior, where bees conduct different activities according to their respective division of labor, and realize the sharing and communication of swarm information, so as to find the optimal solution to the problem. The teaching and Learning Optimization (TLBO) algorithm^13^ simulates the class-based learning method, and individual students reserve knowledge through two stages: "Teaching" and "Learning", so as to improve the ability of each individual.

In order to meet the requirements of the solution accuracy and speed in the engineering application field, the scholars have made a more profound exploration of the group intelligence evolution algorithm, mainly focusing on the following two aspects:

On the one hand, the existing group intelligence evolution algorithm is improved to further improve its optimization performance. The more representative algorithms are as follows: In 2017, Wei Sun et al. proposed an all-dimensional particle swarm algorithm with randomly selected neighbors^14^ (ADN-RSN-PSO). This algorithm early adopted the randomly selected neighborhood (RSN) learning strategy to improve group diversity, and later adopted the all-dimensional neighborhood (ADN) learning strategy to improve the convergence rate. In 2018, Deng Xianli et al. proposed a multiple group based self-adaptive migration PSO algorithm (MSMPSO)^15^, the algorithm integrates two commonly used neighbor topology to give individuals more information sources, based on the parallel evolution of multiple sub-populations, gives different search characteristics to each sub-population, realize the collaboration and reasonable allocation of sub-population resources, and finally improve the comprehensive performance of the algorithm. In 2019, Gurcan Yavuz and Dogan Aydın proposed an algorithm for artificial swarm based on self-adaptive search equations (SSEABC^)^^16^, the algorithm integrates self-adaptive strategies, local search strategies, incremental population size strategies, and conversion conditions for local and internal search, showing some advantages through comparative experiments. In 2019, Juan Li et al. proposed an improved adaptive knowledge-learning cuckoo search algorithm (I-PKL-CS)^17^, the proposed algorithm introduces a learning model with individual history knowledge and group knowledge in the original CS algorithm, and adopts threshold statistical learning strategies to develop the potential of individual knowledge learning and group knowledge learning, providing a good trade-off for exploration and development. In 2020, an efficient dual-adaptive random standby enhanced whale optimization algorithm (RDWOA)^18^ was proposed by Huiling Chen et al. the algorithm introduces two strategies in the original algorithm: one is random standby or random replacement strategy to improve the convergence speed of the algorithm; the other is to introduce double adaptive weight strategy to improve the overall search ability of the algorithm. In 2021, An improved artificial tree algorithm with two populations (IATTP)^19^ was proposed by Yaping Xiao et al., The algorithm proposes the competition mechanism between populations, and the migration can expand the population, reduces the inefficient population size, and realizes the reasonable interaction between the population and the branches In 2022, Zhongkai Feng et al. proposed an enhanced sine cosine algorithm (ESCA)^20^, the algorithm uses reverse learning strategy to expand the search scope, adaptive evolution strategy to improve global exploration, community search strategy to increase population diversity, and greedy selection strategy guarantees solution quality, thus improving the convergence accuracy of the algorithm.

On the other hand, many new types of group intelligent evolution algorithms with excellent performance have emerged, such as: In 2020, Selim Yilmaz and Sevil Sen proposed the electric fish optimization (EFO) algorithm^21^ according to the way that the electric fish determine the prey orientation and transfer information to each other, the active and passive electric positioning ability of such fish are thought to be able to well balance local and global search. In 2020, a Side-Blotched Lizard Algorithm(SBLA)^22^ was proposed for the polymorphic populations of lizards simulated by Oscar Maciel et al., the algorithm utilizes three operators to embody the lizard state and uses a sub-population management strategy to simulate the variation of each state lizard population over time. In 2021, Zhang Kaifeng et al. inspired by the modern corporate teamwork behavior and proposed a Cooperation Search Algorithm (CSA)^23^, it uses team communication, reflection learning, and internal competition to complete the global optimization during repeated iterations. In 2020, Yiying Zhang and Zhigang Jin were inspired by the group teaching mechanism to propose the Group Teaching Optimization Algorithm (GTOA)^24^ which is different from the TLBO algorithm, which only includes two stages of "Teaching" and "Learning", GTOA includes four phases: Teacher assignment phase, Ability group phase, Teacher phase, and Student phase, it adopts different teaching methods for students with different amounts of knowledge. Experimental results show that the convergence speed and convergence accuracy of GTOA are significantly better than those of PSO^25^, DE^26^, WOA^27^, NNA^28^, SCA^29^ and TLBO^13^.

Extensive experiments show that compared with the classical swarm intelligence evolutionary algorithm, the recently proposed evolutionary algorithm usually has higher convergence accuracy and faster convergence speed. However, for more complex optimization problems, they also inevitably have many defects, such as insufficient population diversity, easy to fall into local optimal. In order to meet the requirements of practical engineering applications, it is necessary to further improve the optimization performance of the proposed evolutionary algorithm, which is bound to become a research hot-spot in the field of evolution and engineering applications in recent years. Based on the above background, this paper only studies GTOA, proposes an improved group teaching optimization algorithm, and further improves its ability to solve complex optimization problems. First, assign teachers independently to each body, replace the original way of sharing the same teacher, increase the evolutionary direction, and expand population diversity. Second, dynamically divide the good group of students and average group students to replace the original fixed distribution model to meet the different needs of different evolutionary stages for convergence speed and population diversity. Third, the student learning phase cancels the self-learning part with weak effect, increases the mutual learning part, and supplements the population diversity. Fourth, for the average group of students, a new sub-space search model is proposed, and the teaching method of teachers is improved to reduce the loss of diversity in the process of population evolution. Fifth, a new population reconstruction mechanism is proposed to increase the possibility of population jumping out of local optimum. Tested on the CEC2013 test set, the results show that IGTOA has certain advantages in convergence speed, convergence accuracy and stability compared with the other five optimization algorithms.The remaining structure of this article is as follows: The “GTOA” Section introduces how the original algorithm GTOA works. In the “IGTOA” Section, the improved algorithm IGATOA is proposed, and its overall structure and improvement methods are elucidated. The “Experiment and analysis” Section will improve the algorithm and the original algorithm and other excellent algorithms, based on the CEC2013 test function simulation experiments, and obtain results. Finally, “Conclusion” Section provides a concluding overview.

## GTOA


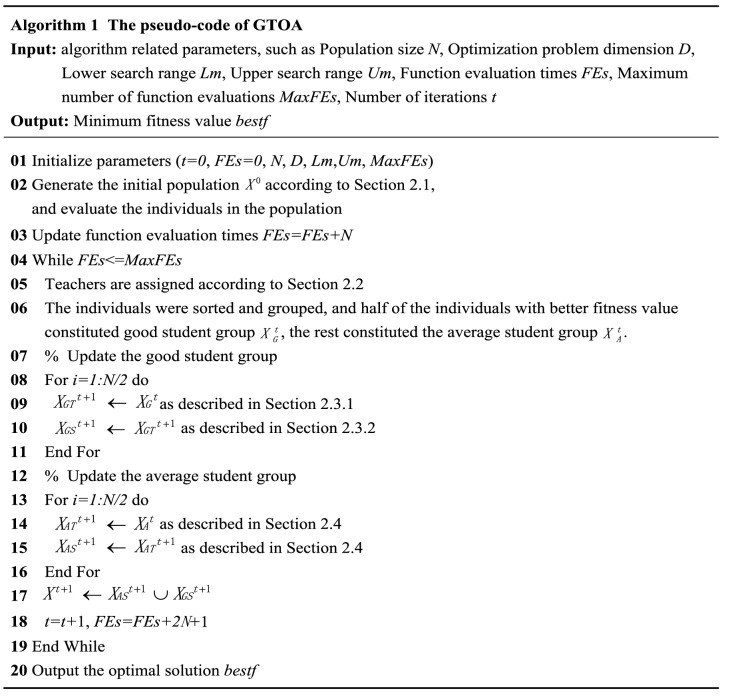


In 2020, Zhang Yiying et al. proposed the GTOA through the simulation group teaching mechanism to solve the continuous function optimization problem. In this algorithm, the decision variable is equal to the discipline, and the individual is equal to the student, that is, the individual student is composed of multiple disciplines, and the fitness value is equal to the knowledge level of the student. For solving the minimization problem, the smaller the fitness value of the individual, the higher the knowledge level of the student. Group the students according to their knowledge level, combine the characteristics of the group, adopt different learning methods, and constantly increase the amount of knowledge of the students in the process of evolution. The pseudo-code of the GTOA is shown in Algorithm 1, and its key operations are described below.

### Population initialization

Assume that the population size is *N*, and the dimension of the problem to be optimized is *D*, the *i*-th individual in the initial population $X^{0} = [X_{1}^{0} \,,\,X_{2}^{0} \,,\,...\,\,,\,X_{N}^{0} ]^{T}$ can be described as $X_{i}^{o} = [x_{i,1}^{0} ,\;x_{i,2}^{0} ,\;...,\;x_{i,D}^{0} ]$(*i* = 1,2,…,*N*; *j* = 1,2,…,*D*), its *j*-th dimension $x_{i,j}$ is randomly generated according to Eq. (1):

1 $$x_{i,j} = rand(0,1) \times (Um - Lm) + Lm$$

Among them, Um and Lm are the upper and lower limits of the search range of the optimization problem, respectively, and rand(0,1) is a random number between 0 and 1.

### Teacher assignment

For the current population $X^{t}$, a teacher is determined according to Eq. (2) to teach students in each iteration.

2 $$T^{t} = \left\{ {\begin{array}{*{20}l} {X_{1}^{t} } \hfill & {,\;\,f\left( {\frac{{X_{1}^{t} + X_{2}^{t} + X_{3}^{t} }}{3}} \right) \ge f(X_{1}^{t} )} \hfill \\ {\frac{{X_{1}^{t} + X_{2}^{t} + X_{3}^{t} }}{3}} \hfill & {,\;f(X_{1}^{t} ) > f\left( {\frac{{X_{1}^{t} + X_{2}^{t} + X_{3}^{t} }}{3}} \right)} \hfill \\ \end{array} } \right.$$

Among them, $X_{1}^{t}$, $X_{2}^{t}$ and $X_{3}^{t}$ are respectively the three students with the best fitness value, and *t* is the current number of iterations.

### Update good student group

The individual update of the good students group needs to go through the teacher teaching phase and the student learning phase in turn, as follows.

#### Teacher teaching phase

Generally, students in good group have strong ability to accept knowledge, and teachers pay attention to improving the average knowledge of the whole group when teaching. In view of this, the GTOA designed the teacher teaching method as shown in Eq. (3).

3 $$X_{{GT_{i} }}^{{t + 1}} \; = X_{{G_{i} }}^{t} + a \times (T^{t} - F \times (b \times M^{t} + c \times X_{{G_{i} }}^{t} ))$$

Among them,$X_{{GT_{i} }}^{{t + 1}} \;$ represents the individual update of student $X_{Gi}^{t}$ after the teacher's teaching; *F* is the teaching factor, with a random value of 1 or 2; *a*, *b*, *c* are random numbers in [0,1], and *b* + *c* = 1; $M^{t}$ is the average subject knowledge of students in the good group in the t iteration process, as shown in Eq. (4).

4 $$M^{t} {\kern 1pt} = {\kern 1pt} \,\frac{1}{N} \times \sum\limits_{{i = 1}}^{N} {X_{{G_{i} }}^{t} }$$

If the knowledge level of students is improved after teaching, the subject knowledge of students should be updated; otherwise, the original students should remain unchanged. The details are shown in Eq. (5).

5 $$X_{{GT_{i} }}^{{t + 1}} \; = \left\{ {_{{XG_{i}^{t} \quad \;\;,\;\;f(X_{{GT_{i} }}^{{t + 1}} \;) \ge f(X_{{Gi}}^{t} )}}^{{X_{{GT_{i} }}^{{t + 1}} \;\;,\;\;f(X_{{GT_{i} }}^{{t + 1}} \;) < f(X_{{Gi}}^{t} )}} } \right.$$

#### Student learning phase

According to Formula (6), students learn from each other within the group to acquire new knowledge of each subject, and decide whether to update the current individual students according to the method described in Eq. (7).

6 $$X_{{GS_{i} }}^{{t + 1}} \; = \left\{ \begin{gathered} X_{{GT_{i} }}^{{t + 1}} \; + e \times (X_{{GT_{i} }}^{{t + 1}} - X_{{GT_{j} }}^{{t + 1}} ) + g \times (X_{{GT_{i} }}^{{t + 1}} - X_{{G_{i} }}^{t} )\;,\;f(X_{{GT_{i} }}^{{t + 1}} ) < f(X_{{GT_{j} }}^{{t + 1}} ) \hfill \\ X_{{GT_{i} }}^{{t + 1}} - e \times (X_{{GT_{i} }}^{{t + 1}} - X_{{GT_{j} }}^{{t + 1}} ) + g \times (X_{{GT_{i} }}^{{t + 1}} - X_{{G_{i} }}^{t} )\;,\;f(X_{{GT_{i} }}^{{t + 1}} ) \ge f(X_{{GT_{j} }}^{{t + 1}} ) \hfill \\ \end{gathered} \right.$$

7 $$X_{{GS_{i} }}^{{t + 1}} \; = \left\{ {_{{X_{{GS_{i} }}^{{t + 1}} \;\quad \;,\;f(X_{{GT_{i} }}^{{t + 1}} ) \ge f(X_{{GS_{i} }}^{{t + 1}} )}}^{{X_{{GT_{i} }}^{{t + 1}} \;\quad \;,\;f(X_{{GT_{i} }}^{{t + 1}} ) < f(X_{{GS_{i} }}^{{t + 1}} )}} } \right.$$

Among them, *e* and *g* are two random numbers within [0,1]; $X_{{GS_{i} }}^{{t + 1}}$ is the individual updated by $X_{{GT_{i} }}^{{t + 1}} \;$ after learning through the student phase during the *t* + 1 iteration; $X_{{GT_{j} }}^{{t + 1}} \;$ is another individual student randomly selected in this group, and *j ≠ i*.

### Update average student group

Similar to the good group, the individual renewal of the students in the average group also needs to go through the teaching phase and the learning phase successively. Among them, the learning phase of the students in the average group is exactly the same as that of the good group. And according to the differences in the knowledge level of the two groups, the teaching phase of the teachers is different, which is as follows.

In view of the relatively poor knowledge level of students in the average group, teachers are more inclined to improve the knowledge level of individual students in the learning process. The GTOA has developed a teaching plan for the students in the average group, such as Eq. (8). Similar to the teaching of good group teachers, after the teaching, they should also judge whether to update their existing subject knowledge according to Eq. (5).

8 $$X_{AT_{i}}^{t + 1} = X_{A_{i}}^{t} + 2d \times (\,T^{t} - X_{A_{i}}^{t} )$$

Among them, *d* is a random number in the range of [0,1]; $X_{AT_{i}}^{t + 1}$ is the individual student $X_{AT_{i}}^{t + 1}$ updates by learning from the teacher $T^{t}$ during the *t* + 1 iteration.

## IGTOA

**Figure 1:**
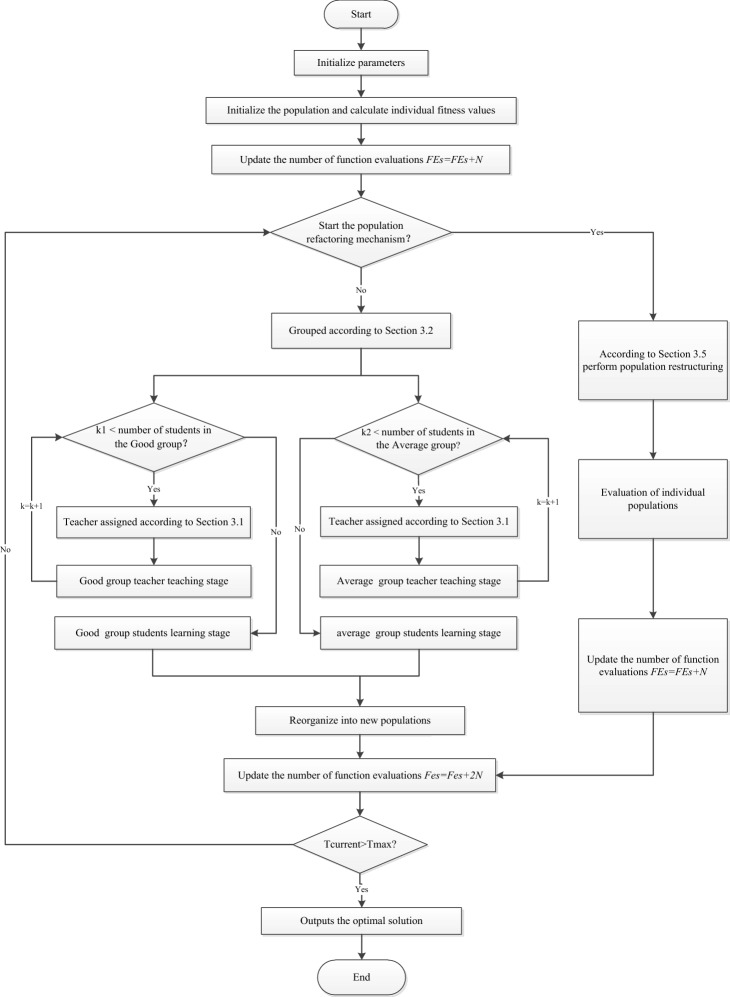
IGTOA flow chart.

A large number of experiments show that for more complex function problems, similar to other swarm intelligence evolutionary algorithms, GTOA also has shortcomings such as slow convergence speed and easy to fall into local optimum. This paper deeply analyzes the reasons for the above problems, and proposes an improved group teaching optimization algorithm (IGTOA), the flow chart of which is shown in Fig. 1.

### Teacher assignment

As described in “Teacher assignment” Section in the teacher assignment stage of the GTOA, from the optimal individual and the average of the three better individuals, the winner is selected as the teacher of all students, and all students in the subsequent teaching stage of the good group and the teaching phase of the average group are selected. Students learn only from that teacher. Obviously, such a single-teacher learning mode will make individuals approach to it quickly, resulting in a single direction of individual exploration, rapid decline of population diversity, and easy to fall into local optimal. In order to solve the above problems, in IGTOA, all students independently select teachers for learning in the following way, as shown in Eq. (9).

9 $$T^{\,t} \equiv \left\{ \begin{gathered} X_{1}^{t} \quad \quad \quad \quad \quad \quad ,\;if\;rand < P\_teacher \hfill \\ \frac{{X_{1}^{t} + X_{2}^{t} + X_{3}^{t} }}{3}\quad \;,\;otherwise \hfill \\ \end{gathered} \right.$$

Among them, $T^{t}$ represents the teacher assigned to the students during the teacher teaching stage; *P*_*_teacher*_ represents teacher assignment probability. Generally, *P*_*_teacher*_ = 0.5 can achieve better results.

By the Eq. (9) can be seen, each individual is independent of choosing the best individual and one of the three is the center of the optimal individual as a teacher, and learn from it, evolutionary direction is no longer a single individual, can better maintain the population diversity, and the best individual and all three is the center of the optimal individual carries the better the evolution of the information, will not be too much lower convergence speed. In short, this new allocation method of independent teachers can balance the convergence rate and population diversity.

### Adaptive grouping

In GTOA, according to the fitness of individuals, half of the individuals with better fitness value in the whole population are divided into good group, and the rest are divided into average group. Through the overall analysis of GTOA, it can be found that the evolution of average students is relatively slow, and the main function is to provide evolutionary information for good students to explore and develop new positions, so that they can quickly approach the global optimal position. In short, the average group focused on maintaining population diversity, while the good group was mainly responsible for exploration and search.

**Figure 2:**
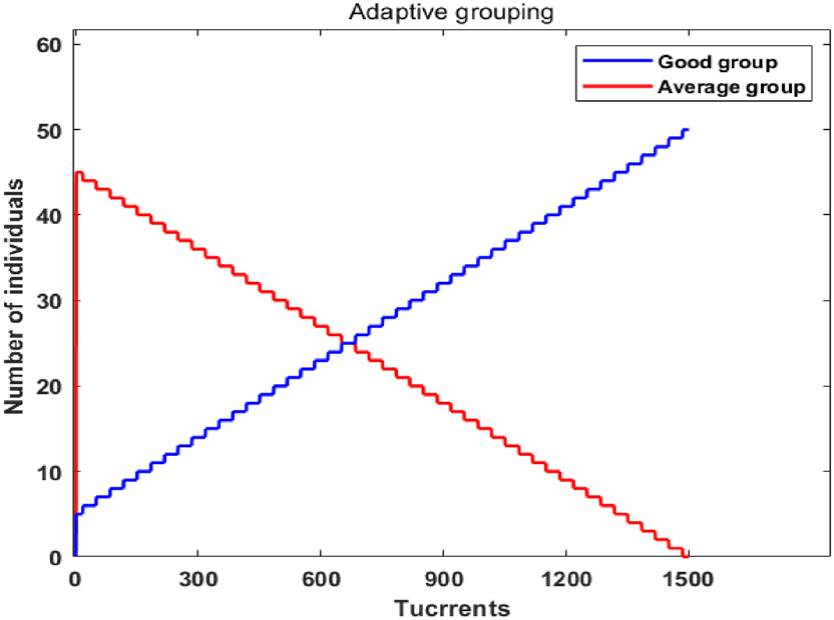
Individual number of students in the two groups changed.

Generally, different evolutionary stages have different requirements for algorithm performance: In the early stage of iteration, the fitness value gap between individuals is large, and the population diversity is good. Usually, it is hoped that the algorithm will quickly converge to the region where the optimal solution is located. As the evolution progresses, the fitness value gap between individuals is decreasing. , the individuals become more and more similar, and the population diversity gradually deteriorates. It is expected that the algorithm can increase the population diversity in order to have the ability to jump out of the local optimum. In order to better meet the needs of different evolutionary stages of the algorithm, in the early stage of evolution, the scale of the average group should be appropriately reduced and the scale of the good group should be expanded; and vice versa. Based on the above ideas, this paper proposes a dynamic allocation method of the number of good group and average group students as shown in Eq. (10), and the allocation process is shown in Fig. 2.

10 $$\left\{ \begin{gathered} N_{{\hbox{A}}}^{t} = P\_group \times N + \,\left\lfloor {N - 2 \times P\_group \times N} \right\rfloor \times \frac{t}{{\max \_t}} \hfill \\ N_{{\hbox{G}}}^{t} = N - N_{{\hbox{A}}}^{t} \hfill \\ \end{gathered} \right.$$

Among them, *N*_*A*_^*t*^ and *N*_*G*_^*t*^ represent the number of students in the average group and the good group in the *t*-th iteration respectively; *P*_*_group*_ is the grouping ratio, generally, *P*_*_group*_ = 0.1 can achieve better results; $\left\lfloor \bullet \right\rfloor$ represents rounding; *t* is the current iteration number of times; *max_t* maximum number of iterations.

As can be seen from Fig. 2, in the process of 1500 iterations, the number of students in the good group was significantly higher than that in the average group in the early stage of evolution. With the increase of iterations, the number of students in the good group gradually decreased, in line with the different needs of good group size and average group size in different evolutionary stages.

### Improvement of update method for good group in student learning phase

GTOA divides the population into good and average groups according to the merits of the fitness value, and each group goes through the teacher teaching phase and the student learning phase accordingly, and the two groups share the same student learning strategy. Unlike the teacher phase, the student learning phase does not learn from good teachers, but from other individuals in the group and themselves. Therefore, the student learning phase is mainly responsible for maintaining the population diversity within the group. An in-depth analysis of the way in which individuals are updated at the student learning stage as shown in Eq. (6) shows that the new individual is actually constituted by "Original individual + Mutual learning + Self-learning". Among them, the mutual learning part randomly selects other individuals in the group to learn, which can provide population diversity to a certain extent; the self-learning part is that the current individual learns from the individual who has not undergone the teacher stage, if the individual is not retained after going through the teacher stage, the current individual is exactly the same as the individual who experienced the teacher stage before. Obviously, self-learning is completely meaningless, and if the individual is retained after going through the teacher stage, the current individual is better than the individual before the teacher stage, and it is difficult to learn from it to produce new individuals who are better than themselves, then the self-learning stage will still not play a role. In short, the self-learning component of GTOA is extremely weak. In order to further enhance its population diversity, the self-learning part is abolished, learning from other individuals is added, and the new student learning stage individual renewal method is proposed as shown in Eq. (11).

11 $$X_{{GS_{i} }}^{{t + 1}} = X_{{GT_{i} }}^{{t + 1}} + a \times (X_{{GT_{j} }}^{{t + 1}} - X_{{GT_{i} }}^{{t + 1}} ) + b \times (X_{{GT_{k} }}^{{t + 1}} - X_{{GT_{i} }}^{{t + 1}} )$$

where $X_{{GS_{j} }}^{{t + 1}} ,X_{{GT_{k} }}^{{t + 1}}$ are two different individuals randomly selected in the good group, *i* ≠ *j* ≠ *k*, respectively, and the definitions of parameters *a* and *b* are shown in Eq. (12).

12 $$\begin{gathered} a = rand \times \text{sgn} (f(X_{GT_{i}}^{{t + 1}} ) - f(X_{GT_{j}}^{{t + 1}} )) \hfill \\ b = rand \times \text{sgn} (f(X_{GT_{i}}^{{t + 1}} ) - f(X_{GT_{k}}^{{t + 1}} )) \hfill \\ \end{gathered}$$

where, ${\text{sgn}} ( \bullet )$ is a symbolic function.

### Improvement of update method for average student group

As described in “Adaptive grouping” Section, the good group in GTOA is mainly responsible for exploration and search, while the average group focuses on maintaining population diversity. Similar to the updating method of students in the good group, students in the average group also conduct a complete search in the D-dimensional search space. Such a wide range of communication in the search space is very likely to make the population quickly close to several superior individuals and gather in a certain area or several regions, resulting in a serious loss of population diversity. If the average group of students does not conduct a complete large-scale search in the D-dimensional search space, but only conducts a small-scale search in some dimensions, it is easy to overcome the above shortcomings.

Based on the above analysis, average group of students in teachers' teaching and students' learning phase are small range subspace search, among them, the average group of students to study with good group of students learning phase in the same way, and the new design of ordinary teachers teaching phase and sub-space model way choice of dimension to search in the specific as follows.

#### Dimension selection in subspace patterns

In order to maintain the diversity of the population as much as possible, in the subspace search mode we designed for the average group of students to update, the number of dimensions to be updated by each individual and the specific dimensions are randomly generated, as follows: First, for each individual, a random integer k is randomly generated in [1,D], which is the total number of dimensions that the individual needs to update; Then, k random integers are randomly generated in the dimension space [1,D], and the subsequent subspace search will be performed in its corresponding dimension.

**Figure 3:**
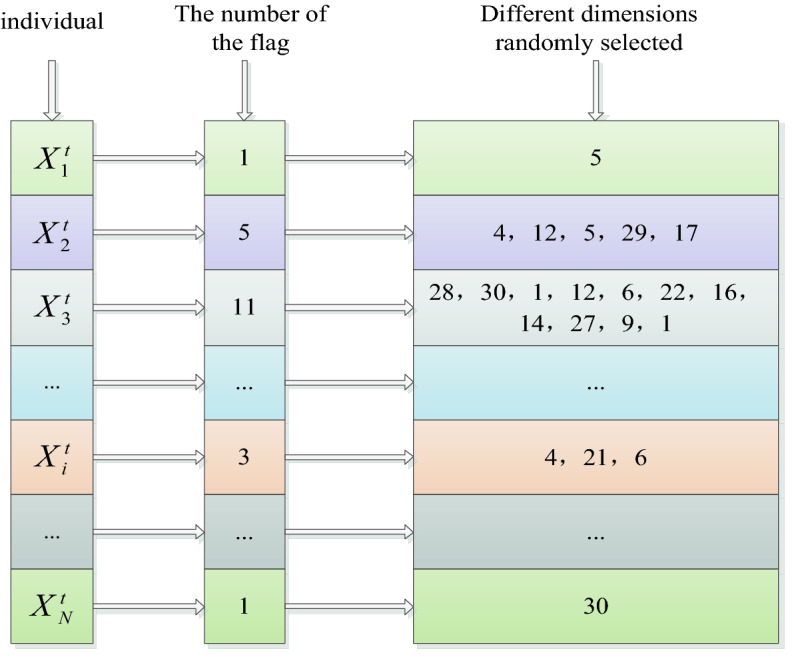
Dimension selection in subspace search mode.

To further understand the dimension selection in the above subspace pattern, a concrete example is shown in Fig. 3. Given that the dimension of the problem to be optimized is 30, for example, for individual X2, the number of randomly generated dimensions to be updated is 5, and 5 random integers are randomly generated within [1, 30], which are {4,12,5,29,17} respectively, indicating that individual X2 will only search on the dimension {4,12,5,29,17} when conducting subspace search, the other dimensions don't change.

#### Improvement of teacher teaching phase

It can be seen from Algorithm 1 that the students in the good group and the average group in GTOA only rely on the students in this group to update their subject knowledge. When the next iteration is regrouped, the good group and the average group can exchange information with each other. However, since the students in the good group are better than the students in the average group, after the knowledge update, the students in the good group are basically better than the students in the average group. Therefore, even after regrouping, only a very small number of students from the average group entered the good group. Obviously, students in the good group only absorbed a very small amount of the evolutionary information of the average group, and did not really get the diversity supplement. Basically, they still relied on their original evolutionary information to evolve, which was easy to quickly converge to a local optimum. In order to make the good group get the full diversity supplement, it is necessary to further improve the subject knowledge of the students in the average group and increase the opportunity for them to enter the good group. In view of this, the teaching methods of average group teachers are improved as follows, as shown in Eq. (13).

13 $$X_{AT_{i,j}}^{t + 1} = X_{A_{i,j}}^{t} + F_{1} \times ((a \times T_{j}^{t + 1} + (1 - a) \times X_{GT_{m,j}}^{t + 1} ) - X_{A_{i,j}}^{t} )$$

where $T{\kern 1pt}_{j}^{t + 1}$ represents the teachers assigned according to Eq. (10), and one teacher is independently selected from each dimension; $X_{GT_{m,j}}^{t + 1}$ represents an individual selected randomly from $X_{GT}^{t + 1}$, and likewise, an individual selected independently from each dimension; *F*_*1*_ is the random number between [1, 2], and *a* is the random number between [0,1].

A comparison of Eqs. (8) and (13) can be found that: First, in the GTOA, the learning objects in the average group are all teachers. In this section, all the students of the teachers in the good group are also listed as the learning objects. Because the good group students are the result of learning from teachers, the gap between the average group and the good group is further shortened, and the possibility of the average group gene flowing into the good group is increased. Second, compared with only learning from teachers themselves, the new teaching method of teachers has more combinations of learning objects, which greatly improves the diversity of students in the average group. Although the evolutionary information of some outstanding group students will be mixed into the average group students, the evolutionary genes flowing in different dimensions come from different outstanding students, and the genes of the average group students are completely preserved in the dimension without subspace search, so that there is a big difference between the evolutionary information of the average group students and the evolutionary information of the good group students. Therefore, when the students of the average group flow into the good group, they can be supplemented with a certain population diversity. To sum up, the new teaching method of average group teachers proposed in this section has certain advantages.

**Figure 4:**
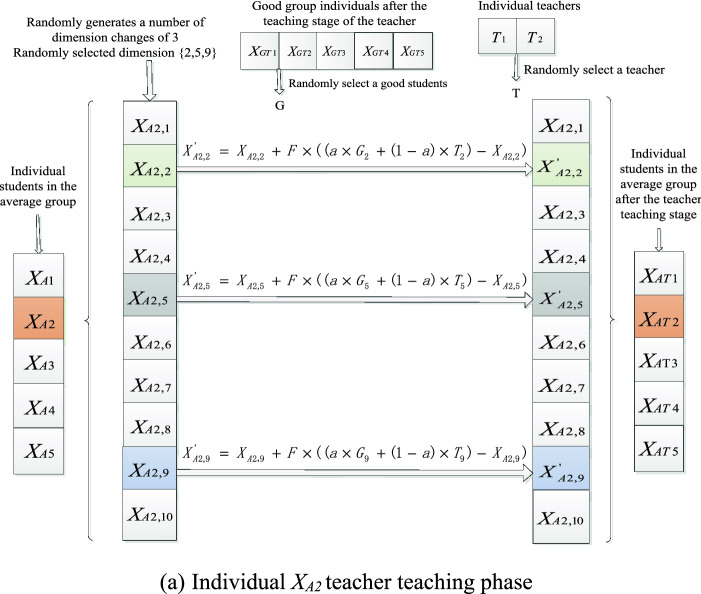
Subspatial learning mode of teacher teaching stage of average group individual *X*_*A2*_.

In order to further understand the subspace search mode of teacher teaching phase and student learning phase in the average group, a specific example is given in Fig. 4. Given that the dimension of optimization problem is 10 and the number of individuals is 5. In the teacher teaching phase, the number of dimensions to be updated in the randomly generated subspace of individual XA2 is 3, and 3 dimensions are randomly selected for subspace search, including {2, 5, 9}, and other dimensions remain unchanged. For dimension 2, random teacher T1 and good group student XG2 learn from their 2-nd dimension according to formula (13); for dimension 5, teacher T2 and the 5th dimension of good group students XG3 were randomly selected for learning; and for dimension 9, the teacher T1 and the 9-th dimension of good group XG4 were randomly selected for learning. Assuming that the newly generated individual is superior to the original, the original individual will update its knowledge of each subject, otherwise unchanged.

It should be noted here that the students in the average group adopt the same learning method as the good group students in the learning stage, as shown in Eq. (11). The difference is that they do not use the full space search mode, but use the subspace search mode.

### Population reconstruction mechanism

A large number of experimental studies have shown that, similar to other swarm intelligence evolutionary algorithms, for very complex optimization problems, with the progress of evolution, GTOA also has the defects of slow evolution and difficulty in obtaining the global optimal solution. In order to solve this problem, this section proposes a new population reconstruction mechanism, which mainly includes the starting conditions of the population reconstruction mechanism and the population reconstruction strategy. The details are as follows.

#### Initiating conditions of population reconstruction mechanism

As we all know, swarm intelligence evolutionary algorithm will show the following obvious characteristics when it evolves slowly: in the process of successive iterations, the optimal value obtained by the population does not change. Therefore, this excerpt uses the change of global optimal value as the starting condition of population reconstruction mechanism. Specific methods are as follows: Initialization parameters *change_flag* = 1 and *flag,* where *change_flag* is used to record the number of times that the global optimal value does not change continuously, *flag* is the threshold that determines the local optimal value; Then, the Euclidean distance of the globally optimal individual in two consecutive iterations is calculated according to Eq. (14), if *move* = 0, *change_ flag* = *change_ flag* + 1, otherwise, *change_ flag* = 1. When *change_ flag* = *flag*, the population reconstruction policy is started and *change_ flag* is set to 1.

14 $$move = \sqrt {\sum\limits_{i = 1}^{D} {(bestX^{t} (i) - bestX^{t - 1} (i))^{2} } }$$

Among them, $bestX^{t}$ and $bestX^{t - 1}$ represent the optimal individuals in the *t*-th and (*t*-1)-th iterations, respectively.

#### Population reconstruction strategy

Experimental results show that for complex optimization problems, the improved algorithm proposed in this paper has a good ability to maintain population diversity. Even if the phenomenon of slow evolution occurs, the individual differences in the current population are relatively obvious, and the clustering and high similarity of individuals are not presented. Through in-depth analysis of the overall optimization process of GTOA, it is not difficult to find the essential reasons for the above phenomenon as follows: At the beginning of its evolution, GTOA started from a very limited number of individuals. Under the guidance of excellent teachers, it drove each individual to move from the area to the better area, and gradually narrowed the search range, so that outstanding individuals could use the help of a smaller range. Other individuals conduct further in-depth and refined exploration, thereby stimulating better evolutionary information to improve teachers. However, for very complex optimization problems, there are often multiple local optimal solutions, and the global optimal solution is hidden in a narrow region. In the huge search space, each individual will be drawn to several local optimal regions with a high probability, because the local optimal values are relatively similar, it is difficult for each individual to jump out of the local optimal region. Although good population diversity can be maintained, the search area cannot be reduced, thus it is difficult to provide motivation for teachers to further fine exploration. To sum up, in order to force teachers to have the motivation to search carefully, the search scope must be appropriately narrowed and other evolutionary information must be introduced.

**Figure 5:**
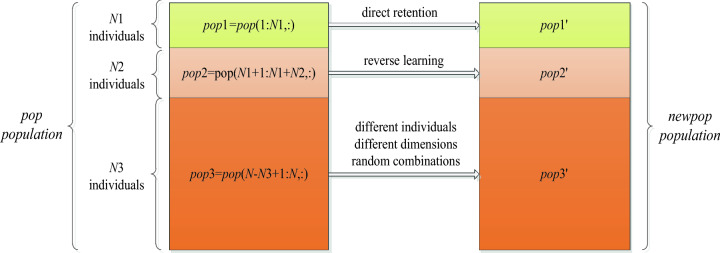
Population reconstruction mechanism.

Generally, GTOA in slow evolution of several iterations, the better individual has acquired when the area before the local optimum, obviously, compared with extensive search in large search space, surrounded by individual only in multiple local optimal point exploration of smaller scope, it is easier to search the global optimal peak of the narrow scope. In addition, references ^30^ and ^31^ have known that the reverse learning strategy of replacing the original candidate solution by the relative points of the candidate solution is a better estimation of the original candidate solution, and generally achieves better optimization results compared with the method of replacing the original candidate solution by random points. Based on the above reasons, a new population reconstruction method is proposed in this section, as shown in Fig. 5, as follows: Firstly, the population was divided into three parts according to fitness, including *pop*1, *pop*2 and *pop*3, and the numbers of sub-populations were *N*1 = 0.1* N*, *N*2 = 0.4* N* and *N*3 = 0.5* N*, respectively. Then, the individuals in the sub-population *pop*1 are retained directly as *pop*1', individuals in the sub-population of *pop*2 randomly choose the dimensions to learn backwards to form the *pop*2' according to the Eq. (15), and the individuals in the population *pop*3 are combined into new individuals by randomly selecting different dimensions of the individuals in *pop*1 according to formula (16), thereby forming *pop*3'. Finally, sub-populations *pop*1', *pop*2' and pop3' were merged to form a new population, *newpop*, to participate in the next iteration. It should be noted that the reconstructed population does not preferentially retain the original population, but directly participate in the evolution of the next generation.

15 $$pop2_{i,j}^{^{\prime}} = \frac{Um - Lm}{{2l}} - \frac{{pop2_{i,j} }}{2l}\quad ,\quad i \in \left[ {1\,,N2} \right]$$

where *l* is the scaling coefficient of the lens, typically, *l* = 10.

16 $$pop3_{i,j}^{^{\prime}} = pop1_{k,j}^{{}} \quad ,\;i \in \left[ {{\kern 1pt} 1\,,N3} \right]$$

where *k* is a random integer of [1, *N*1].

The above population reconstruction methods have the following advantages: First, in several iterations, although the optimal value has not changed, sufficient communication has been carried out between individuals, and effective evolutionary information in poor individuals has been contained in excellent individuals. Therefore, retaining some excellent individuals and removing half of the poor individuals can basically represent the current evolutionary information and will not affect the exploration ability of the population. Second, the reverse learning is carried out on the individuals in the sub-population pop2, which not only further effectively narrows the search range, but also introduces other evolutionary information, which further provides the impetus for the evolution of outstanding individuals. Third, the individual parts and even all dimensions of the population pop3' are randomly selected from the best part of the individuals, and obviously, they all belong to the partially sampled individuals in the region formed by the best part of the individuals. It not only effectively reduces the search area, but also because they are the recombination of the various dimensions of the better individuals, although they contain part of the evolutionary information of the better individuals, they are quite different from them, which supplements the population diversity to a certain extent, so that the algorithm has the power to further fine-tune the search.

**Figure 6:**
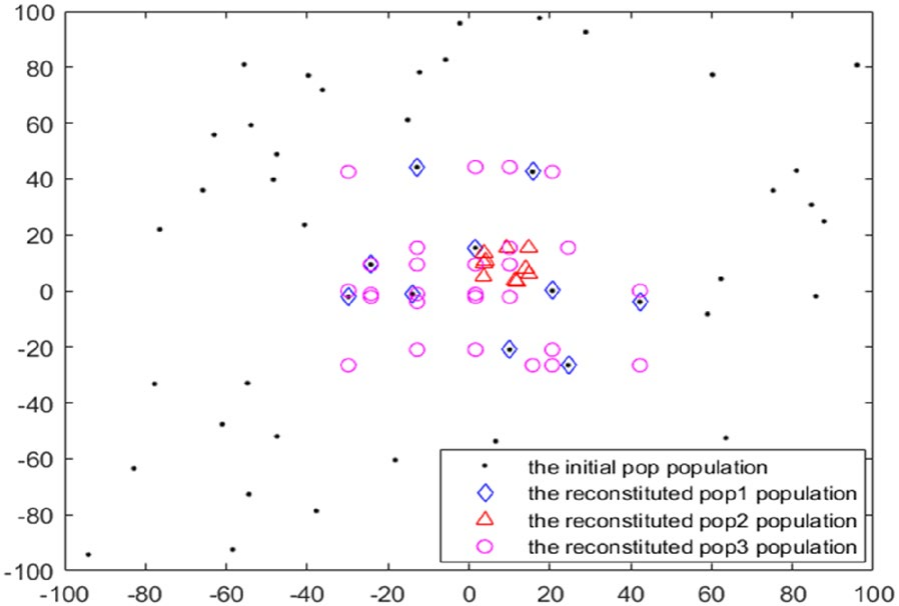
Comparison of individual distribution before and after population reconstruction.

Figure 6 shows the comparison before and after population reconstruction. Assuming that the number of individuals is 50, the problem to be optimized is a sphere function with a dimension of 2. It can be seen that the search range is significantly reduced after population reconstruction, and it is closer to the global optimum.

### The complexity analysis of the algorithm

The population size of IGTOA algorithm is *N*; the number of students in excellent and ordinary groups is *N*_*good*_ and *N*_*average*_ respectively; and the problem dimension is *D*. As shown in Fig. 1, the IGTOA algorithm includes the following four main steps: good group teacher stage (*T*_*_gt*_), good group student stage (*T*_*_gs*_), general group teacher stage (*T*_*_at*_), general group student stage (*T*_*_as*_). The time overhead of the IGTOA algorithm also mainly comes from these four operations. At each generation of the IGTOA algorithm runs, the worst-time complexity of the above four operations is analyzed as follows:

The good group teacher stage (*T*_*_gt*_) needs to calculate *N*_*good*_ × *D* times at most formula (3), so its corresponding worst time complexity is O(*N*_*good*_ × *D*); the good group student stage (*T*_*_gs*_) needs to calculate *N*_*good*_ × *D* times at most formula (11), so its corresponding worst time complexity is O(*N*_*good*_ × *D*); the average group teacher stage (*T*_*_at*_) needs to calculate *N*_*average*_ × *D* times formula (13) at most, so its corresponding worst time complexity is O(*N*_*average*_ × *D*); the average group student stage (*T*_*_as*_) needs to calculate *N*_*average*_ × *D* times formula (11) at most, so its corresponding worst time complexity is O(*N*_*average*_ × *D*).

Therefore, the worst time complexity required for each generation of IGTOA running in the evolutionary process should be O(*N*_*good*_ × *D*) + O(*N*_*good*_ × *D*) + O(*N*_*average*_ × *D*) + O(*N*_*average*_ × *D*)) ≈O(2* N* × *D*).

## Experiment and analysis

In this part, we test the performance of the proposed IGTOA algorithm through four experiments: the first is the parameter sensitivity analysis; the second is the effectiveness of each improvement strategy; and the third is the performance comparison with other excellent intelligent optimization algorithms. The fourth is to compare the results of each algorithm in engineering applications.

“Effect of the parameters on the algorithm”, “Proof of the effectiveness of the improvement measures”, “Compared with other excellent algorithms” Sections is tested on a CEC2013 test set containing 28 functions. According to the characteristics of these functions, they can be divided into three groups: the first group is the unimodal function F1-F5, the second group is the multimodal function F6-F20, and the third group is the combination function F21-F28. Detailed information about this test set can be found in the literature^32^. Meanwhile, the algorithms are implemented in Matlab 2021a. All the tests are conducted on a personal computer equipped with a core i7-11800H CPU (2.30 GHz) and a 16.0-GB memory.

### Effect of the parameters on the algorithm

The proposed algorithm IGTOA adds to the original GTOA algorithm with the following parameters: including *change_flag*, *Pg*, *l*, *N*1 and *N*2. When examining the influence of one of the above parameters on the performance of the IGTOA algorithm, the parameter is set to 5 sets of values, and the other parameters remain unchanged. In all experiments, the population size *N* = 50, the problem dimension *D* = 30, the maximum function evaluation number *MaxFE*s = 5000*D, and the other parameters were set as follows:

1. When examining the effect of change_flag on the performance of the IGTOA algorithm, *change_flag* is set to 10, 30, 50, 70 and 100, respectively. Other parameters are set as follows: *Pg* = 0.1, *l* = 10, *N*1 = *N*2 = 0.2*N.
2. In the new improvement strategy, the number of people in the average group should be greater than 2, so the minimum *Pg* should be 0.06. When examining the effect of *Pg* on the performance of the IGTOA algorithm, *Pg* was set to 0.06, 0.1, 0.4, 0.7, and 0.9, respectively. Other parameters are set as follows: *change_flag* = 30, *l* = 10, *N*1 = *N*2 = 0.2*N.
3. When examining the effect of l on the performance of the IGTOA algorithm, *l* was set to 5, 10, 20, 30, and 50, respectively. Other parameters are set as follows: *change_flag* = 30, *Pg* = 0.1, *N*1 = *N*2 = 0.2*N.
4. When examining the effect of *N*1 and *N*2 on the performance of IGTOA algorithm, *N*1 and *N*2 are set to the following five values: 0.1*N and 0.2*N, 0.2*N and 0.2*N, 0.3*N and 0.2*N, 0.2*N and 0.1*N, 0.2*N and 0.4*N. Other parameters are set as follows: *change_flag* = 30, *Pg* = 0.1, *l* = 10.

**Figure 7:**
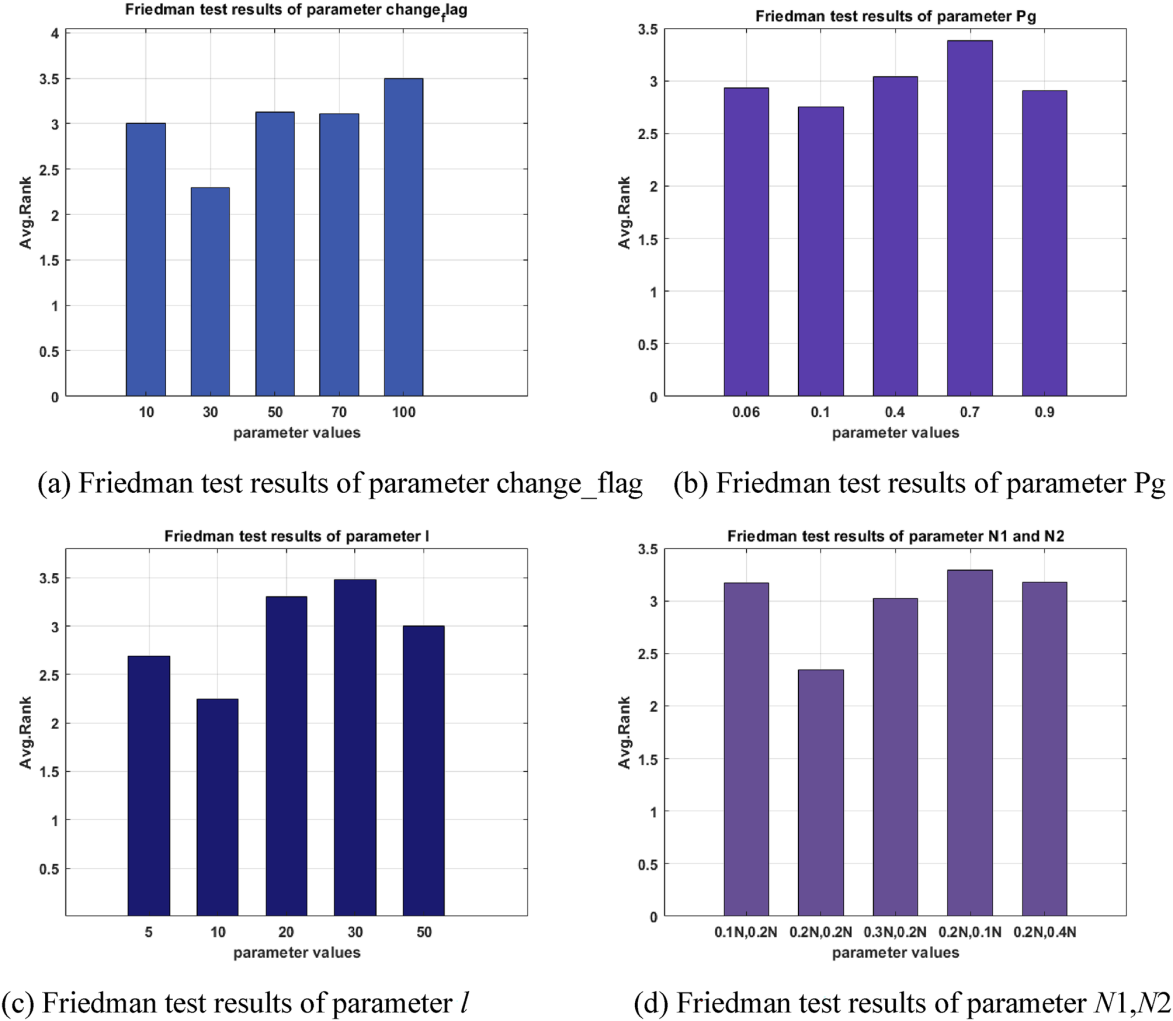
Results of the Friedman test for each parameter.

All of the above experiments were run independently 30 times on the CEC2013 test set, and the average value of the optimal value obtained from each independent run when the same number of preset function evaluation times was reached was counted. The specific data are shown in Tables 1 and 2. In Tables 1 and 2, the parameters that perform best on each function are shaded, and the number of functions is calculated on the last line. In order to further compare the performance of the algorithm, the parameters of the data in Tables 1 and 2 are shown as in Fig. 7, in which the height of the bar graph represents the size of the average rank of each algorithm, the higher the bar graph, the higher the average rank and the higher the rank, the overall performance of the algorithm of the parameter at the value.

**Table 1:** Effect of parameters *change_flag* and *Pg* on IGTOA.

Function	*change_flag*					*Pg*				
	10	30	50	70	100	0.06	0.1	0.4	0.7	0.9
F1	**0.00E + 00**	**0.00E + 00**	**0.00E + 00**	**0.00E + 00**	**0.00E + 00**	**0.00E + 00**	**0.00E + 00**	**0.00E + 00**	**0.00E + 00**	**0.00E + 00**
F2	7.58E + 05	**7.42E + 05**	8.06E + 05	8.07E + 05	8.84E + 05	8.26E + 05	**7.42E + 05**	9.35E + 05	1.22E + 06	2.17E + 06
F3	**0.00E + 00**	**0.00E + 00**	**0.00E + 00**	**0.00E + 00**	**0.00E + 00**	**0.00E + 00**	**0.00E + 00**	**0.00E + 00**	**0.00E + 00**	**0.00E + 00**
F4	**4.76E + 03**	4.79E + 03	7.54E + 03	7.99E + 03	1.04E + 04	5.64E + 03	**4.79E + 03**	7.32E + 03	8.12E + 03	7.55E + 03
F5	**0.00E + 00**	**0.00E + 00**	**0.00E + 00**	**0.00E + 00**	**0.00E + 00**	**0.00E + 00**	**0.00E + 00**	**0.00E + 00**	**0.00E + 00**	**0.00E + 00**
F6	3.18E + 01	**2.44E + 01**	3.68E + 01	2.88E + 01	3.08E + 01	**1.94E + 01**	2.44E + 01	2.47E + 01	3.27E + 01	2.97E + 01
F7	**0.00E + 00**	**0.00E + 00**	**0.00E + 00**	**0.00E + 00**	**0.00E + 00**	**0.00E + 00**	**0.00E + 00**	**0.00E + 00**	**0.00E + 00**	**0.00E + 00**
F8	**2.10E + 01**	**2.10E + 01**	**2.10E + 01**	**2.10E + 01**	**2.10E + 01**	**2.10E + 01**	**2.10E + 01**	**2.10E + 01**	**2.10E + 01**	**2.10E + 01**
F9	**0.00E + 00**	**0.00E + 00**	**0.00E + 00**	**0.00E + 00**	**0.00E + 00**	**0.00E + 00**	**0.00E + 00**	**0.00E + 00**	**0.00E + 00**	**0.00E + 00**
F10	2.50E − 01	2.13E − 01	2.16E − 01	**2.06E − 01**	2.28E − 01	2.23E − 01	2.13E − 01	1.98E − 01	1.62E − 01	**1.50E − 01**
F11	**0.00E + 00**	**0.00E + 00**	**0.00E + 00**	**0.00E + 00**	**0.00E + 00**	**0.00E + 00**	**0.00E + 00**	**0.00E + 00**	**0.00E + 00**	**0.00E + 00**
F12	**0.00E + 00**	**0.00E + 00**	**0.00E + 00**	**0.00E + 00**	**0.00E + 00**	**0.00E + 00**	**0.00E + 00**	**0.00E + 00**	**0.00E + 00**	**0.00E + 00**
F13	**0.00E + 00**	**0.00E + 00**	**0.00E + 00**	**0.00E + 00**	**0.00E + 00**	**0.00E + 00**	**0.00E + 00**	**0.00E + 00**	**0.00E + 00**	**0.00E + 00**
F14	**3.99E + 02**	4.32E + 02	5.24E + 02	5.91E + 02	9.13E + 02	3.73E + 02	4.32E + 02	4.15E + 02	2.78E + 02	**2.70E + 02**
F15	6.57E + 03	**6.34E + 03**	6.87E + 03	6.59E + 03	6.82E + 03	6.95E + 03	**6.34E + 03**	6.82E + 03	7.19E + 03	7.05E + 03
F16	2.61E + 00	**2.55E + 00**	2.65E + 00	2.67E + 00	2.64E + 00	2.65E + 00	**2.55E + 00**	2.68E + 00	2.70E + 00	2.56E + 00
F17	**1.13E + 01**	1.70E + 01	1.97E + 01	2.35E + 01	3.11E + 01	1.80E + 01	1.70E + 01	1.24E + 01	1.09E + 01	**1.08E + 01**
F18	1.95E + 02	**1.84E + 02**	**1.84E + 02**	1.87E + 02	1.93E + 02	1.89E + 02	**1.84E + 02**	1.88E + 02	1.87E + 02	1.89E + 02
F19	6.15E + 00	**4.96E + 00**	7.44E + 00	8.02E + 00	1.13E + 01	4.86E + 00	**4.46E + 00**	6.56E + 00	1.08E + 01	9.07E + 00
F20	**0.00E + 00**	**0.00E + 00**	**0.00E + 00**	**0.00E + 00**	**0.00E + 00**	**0.00E + 00**	**0.00E + 00**	**0.00E + 00**	**0.00E + 00**	**0.00E + 00**
F21	**4.00E + 02**	**4.00E + 02**	**4.00E + 02**	**4.00E + 02**	**4.00E + 02**	**4.00E + 02**	**4.00E + 02**	**4.00E + 02**	**4.00E + 02**	**4.00E + 02**
F22	4.43E + 02	**3.57E + 02**	6.87E + 02	9.43E + 02	1.59E + 03	4.37E + 02	3.57E + 02	5.32E + 02	1.06E + 03	**2.47E + 02**
F23	7.16E + 03	**6.48E + 03**	6.88E + 03	6.82E + 03	6.79E + 03	**6.30E + 03**	6.48E + 03	6.95E + 03	7.16E + 03	7.21E + 03
F24	**2.00E + 02**	**2.00E + 02**	**2.00E + 02**	**2.00E + 02**	**2.00E + 02**	**2.00E + 02**	**2.00E + 02**	**2.00E + 02**	**2.00E + 02**	**2.00E + 02**
F25	2.23E + 02	2.22E + 02	2.22E + 02	**2.18E + 02**	2.20E + 02	2.32E + 02	2.22E + 02	2.20E + 02	**2.18E + 02**	2.19E + 02
F26	2.77E + 02	**2.68E + 02**	2.72E + 02	2.87E + 02	2.86E + 02	2.83E + 02	**2.68E + 02**	2.80E + 02	2.83E + 02	2.77E + 02
F27	**1.61E + 03**	**1.61E + 03**	**1.61E + 03**	**1.61E + 03**	**1.61E + 03**	**1.61E + 03**	**1.61E + 03**	**1.61E + 03**	**1.61E + 03**	**1.61E + 03**
F28	8.81E + 02	8.67E + 02	8.62E + 02	**8.61E + 02**	8.66E + 02	8.42E + 02	8.67E + 02	**8.32E + 02**	8.52E + 02	8.49E + 02
	16	22	14	16	13	15	20	14	14	17

Note: The parameters that perform best on each function are bolded.

**Table 2:** Effect of parameters *l* and *N*1, *N*2 on IGTOA.

Function	*l*					*N1, N2*				
	5	10	20	30	50	0.1 N 0.2 N	0.2 N 0.2 N	0.3 N 0.2 N	0.2 N 0.1 N	0.2 N 0.4 N
F1	**0.00E + 00**	**0.00E + 00**	**0.00E + 00**	**0.00E + 00**	**0.00E + 00**	**0.00E + 00**	**0.00E + 00**	**0.00E + 00**	**0.00E + 00**	**0.00E + 00**
F2	**7.27E + 05**	7.42E + 05	8.85E + 05	8.51E + 05	8.99E + 05	8.08E + 05	**7.42E + 05**	7.69E + 05	8.17E + 05	7.91E + 05
F3	**0.00E + 00**	**0.00E + 00**	**0.00E + 00**	**0.00E + 00**	**0.00E + 00**	**0.00E + 00**	**0.00E + 00**	**0.00E + 00**	**0.00E + 00**	**0.00E + 00**
F4	5.57E + 03	**4.79E + 03**	5.66E + 03	6.62E + 03	6.37E + 03	5.17E + 03	**4.79E + 03**	6.25E + 03	4.86E + 03	8.55E + 03
F5	**0.00E + 00**	**0.00E + 00**	**0.00E + 00**	**0.00E + 00**	**0.00E + 00**	**0.00E + 00**	**0.00E + 00**	**0.00E + 00**	**0.00E + 00**	**0.00E + 00**
F6	3.69E + 01	**2.44E + 01**	2.59E + 01	2.49E + 01	4.47E + 01	3.62E + 01	2.44E + 01	3.06E + 01	**2.28E + 01**	3.14E + 01
F7	**0.00E + 00**	**0.00E + 00**	**0.00E + 00**	**0.00E + 00**	**0.00E + 00**	**0.00E + 00**	**0.00E + 00**	**0.00E + 00**	**0.00E + 00**	**0.00E + 00**
F8	**2.10E + 01**	**2.10E + 01**	**2.10E + 01**	**2.10E + 01**	**2.10E + 01**	**2.10E + 01**	**2.10E + 01**	**2.10E + 01**	**2.10E + 01**	**2.10E + 01**
F9	**0.00E + 00**	**0.00E + 00**	**0.00E + 00**	**0.00E + 00**	**0.00E + 00**	**0.00E + 00**	**0.00E + 00**	**0.00E + 00**	**0.00E + 00**	**0.00E + 00**
F10	2.14E − 01	**2.13E − 01**	2.26E − 01	2.31E − 01	1.92E − 01	2.49E − 01	2.13E − 01	**1.99E − 01**	2.79E − 01	2.16E − 01
F11	**0.00E + 00**	**0.00E + 00**	**0.00E + 00**	**0.00E + 00**	**0.00E + 00**	**0.00E + 00**	**0.00E + 00**	**0.00E + 00**	**0.00E + 00**	**0.00E + 00**
F12	**0.00E + 00**	**0.00E + 00**	**0.00E + 00**	**0.00E + 00**	**0.00E + 00**	**0.00E + 00**	**0.00E + 00**	**0.00E + 00**	**0.00E + 00**	**0.00E + 00**
F13	**0.00E + 00**	**0.00E + 00**	**0.00E + 00**	**0.00E + 00**	**0.00E + 00**	**0.00E + 00**	**0.00E + 00**	**0.00E + 00**	**0.00E + 00**	**0.00E + 00**
F14	**3.86E + 02**	4.32E + 02	4.11E + 02	4.68E + 02	5.31E + 02	6.15E + 02	4.32E + 02	4.50E + 02	5.84E + 02	**4.05E + 02**
F15	6.76E + 03	**6.34E + 03**	6.81E + 03	6.64E + 03	6.60E + 03	**5.71E + 03**	6.34E + 03	6.76E + 03	6.48E + 03	6.08E + 03
F16	2.64E + 00	2.55E + 00	2.66E + 00	2.65E + 00	**2.54E + 00**	2.64E + 00	2.55E + 00	2.71E + 00	**2.51E + 00**	2.63E + 00
F17	1.76E + 01	**1.70E + 01**	1.74E + 01	1.75E + 01	2.02E + 01	2.21E + 01	**1.70E + 01**	1.80E + 01	2.12E + 01	1.86E + 01
F18	1.87E + 02	**1.84E + 02**	1.88E + 02	1.93E + 02	1.85E + 02	1.88E + 02	**1.84E + 02**	1.87E + 02	1.90E + 02	1.90E + 02
F19	6.33E + 00	**4.96E + 00**	6.04E + 00	6.08E + 00	5.07E + 00	5.46E + 00	4.96E + 00	6.37E + 00	7.20E + 00	**4.43E + 00**
F20	**0.00E + 00**	**0.00E + 00**	**0.00E + 00**	**0.00E + 00**	**0.00E + 00**	**0.00E + 00**	**0.00E + 00**	**0.00E + 00**	**0.00E + 00**	**0.00E + 00**
F21	**4.00E + 02**	**4.00E + 02**	**4.00E + 02**	**4.00E + 02**	**4.00E + 02**	**4.00E + 02**	**4.00E + 02**	**4.00E + 02**	**4.00E + 02**	**4.00E + 02**
F22	5.46E + 02	**3.57E + 02**	4.35E + 02	4.95E + 02	4.81E + 02	5.54E + 02	**3.57E + 02**	4.83E + 02	5.07E + 02	3.96E + 02
F23	6.89E + 03	**6.48E + 03**	6.86E + 03	6.89E + 03	6.51E + 03	**6.17E + 03**	6.48E + 03	6.70E + 03	6.62E + 03	6.69E + 03
F24	**2.00E + 02**	**2.00E + 02**	**2.00E + 02**	**2.00E + 02**	**2.00E + 02**	**2.00E + 02**	**2.00E + 02**	**2.00E + 02**	**2.00E + 02**	**2.00E + 02**
F25	**2.19E + 02**	2.22E + 02	2.29E + 02	2.23E + 02	**2.19E + 02**	2.27E + 02	2.22E + 02	**2.13E + 02**	2.26E + 02	2.29E + 02
F26	2.73E + 02	**2.68E + 02**	2.87E + 02	2.80E + 02	2.87E + 02	2.77E + 02	**2.68E + 02**	2.83E + 02	2.77E + 02	2.93E + 02
F27	**1.61E + 03**	**1.61E + 03**	**1.61E + 03**	**1.61E + 03**	**1.61E + 03**	**1.61E + 03**	**1.61E + 03**	**1.61E + 03**	**1.63E + 03**	**1.61E + 03**
F28	**8.45E + 02**	8.67E + 02	8.70E + 02	8.75E + 02	8.64E + 02	**8.52E + 02**	8.67E + 02	8.66E + 02	8.74E + 02	9.40E + 02
	17	23	13	13	15	16	19	15	15	15

Note: The parameters that perform best on each function are bolded.

According to the data in Table 1, when *change_flag* = 30, the relatively best results are achieved on the 22 functions; when *change_flag* is 10 or 70, it works relatively best on 16 functions; and when the *change_flag* = 50 or 100, the relatively best results are achieved on 14 and 13 functions, respectively. As can also be seen from the results in Fig. 7a, IGTOA algorithm performance is optimal when *change_flag* = 30, when *change_flag* = 10,50 and 70, IGTOA algorithm performance is little different, but it is inferior to IGTOA algorithm performance when *change_flag* = 30, while when *change_flag* = 100, IGTOA algorithm performance is not satisfactory. In “Conclusion” Section, the IGTOA algorithm is more sensitive to the parameter *change_flag*, and it performs the best when *change_flag* = 30. Analyzing other data in Tables 1 and 2 and Fig. 7 in the same way, we can find that the IGTOA algorithm is sensitive to both parameters *l* and *N*1 and *N*2, with slightly decreased sensitivity to parameter *Pg*, and the IGTOA algorithm performs best when *Pg* = 0.1, *l* = 10 and *N*1 = *N*2 = 0.2*N. To sum up. when the parameters *change_flag*, *Pg*, *l*, *N*1, *N*2 are set to 30, 0.1, 10, and 0.2* N*,0.2* N*, respectively, IGTOA has a good optimization effect. If you want to obtain a better effect for a certain actual optimization problem, the above parameters can also be adjusted several times.

### Proof of the effectiveness of the improvement measures

To verify the effectiveness of each improvement measure in Parts 3.1–3.3, a corresponding improvement strategy was removed in IGTOA, five new improvement algorithms were formed, including the improved algorithm for removing the teacher allocation strategy of “Teacher assignment“ Section in IGTOA, the improved algorithm for removing the adaptive grouping strategy of “Adaptive grouping” Section in IGTOA, the improved algorithm for removing the good group improvement strategy of “Improvement of update method for good group in student learning phase” Section in IGTOA, the improved algorithm for removing the common group improvement strategy of “Improvement of update method for average student group” Section in IGTOA and improved algorithms for the population reconstruction strategy of removing “Improvement of teacher teaching phase” Section in IGTOA. For simplicity, the above five new algorithms are called IGTOA1, IGTOA2, IGTOA3, IGTOA4 and IGTOA5, respectively.

The above five improved algorithms and IGTOA were compared on the CEC2013 test set. To ensure the fairness of the comparison, the parameters of each algorithm are set exactly the same, as follows: population size *N* = 50, problem dimension *D* = 30, maximum evaluation number *MaxFEs* = 5000*D, *change_flag* = 30, *Pg* = 0.1, *l* = 10, *N*1 = *N*2 = 0.2*N. The mean and variance of the optimal values obtained by running the algorithms independently 30 times on each function are counted, and the specific results are shown in Table 3. Among them, the data from outside and in parentheses represent the mean and standard deviation of the optimal values obtained in 30 independent experiments, respectively. The bold value indicates that the performance of the other improved algorithms are significantly worse than IGTOA on the corresponding functions, counting the number of functions for which the improved algorithm is significantly worse than IGTOA in the penultimate row, and giving the average rank and the ranking results of IGTOA and the other five improved algorithms for Friedman test in the last two lines.

**Table 3:** Comparison results of each improvement strategy with IGTOA on the 30-dimensional CEC2013 test suite.

D = 30 (vs. IGTOA)						
Function	IGTOA	IGTOA1	IGTOA2	IGTOA3	IGTOA4	IGTOA5
F1	0.00E + 00 (0.00E + 00)	0.00E + 00 (0.00E + 00)	0.00E + 00 (0.00E + 00)	0.00E + 00 (0.00E + 00)	**2.85E − 27 (8.91E − 27)**	0.00E + 00 (0.00E + 00)
F2	7.42E + 05 (2.91E + 05)	**7.83E + 05 (3.23E + 05)**	**1.08E + 06 (4.55E + 05)**	2.53E + 05 (1.05E + 05)	6.22E + 05 (2.91E + 05)	**7.45E + 05 (2.69E + 05)**
F3	0.00E + 00 (0.00E + 00)	0.00E + 00 (0.00E + 00)	0.00E + 00( 0.00E + 00)	0.00E + 00 (0.00E + 00)	0.00E + 00 (0.00E + 00)	0.00E + 00 (0.00E + 00)
F4	4.79E + 03 (1.51E + 03)	**4.87E + 03 (1.74E + 03)**	**7.42E + 03 (2.23E + 03)**	3.67E + 03 (1.86E + 03)	4.29E + 03 (2.11E + 03)	**1.37E + 04 (3.76E + 03)**
F5	0.00E + 00 (0.00E + 00)	0.00E + 00 (0.00E + 00)	0.00E + 00 (0.00E + 00)	**5.70E − 35 (3.08E − 34)**	**3.08E − 18 (1.37E − 17)**	0.00E + 00 (0.00E + 00)
F6	2.44E + 01 (2.47E + 01)	**3.33E + 01 (2.89E + 01)**	**2.83E + 01 (2.71E + 01)**	**3.79E + 01 (2.90E + 01)**	**3.38E + 01 (2.82E + 01)**	**2.75E + 01 (2.57E + 01)**
F7	0.00E + 00 (0.00E + 00)	0.00E + 00 (0.00E + 00)	0.00E + 00 (0.00E + 00)	0.00E + 00 (0.00E + 00)	0.00E + 00 (0.00E + 00)	0.00E + 00 (0.00E + 00)
F8	2.10E + 01 (4.81E − 02)	2.10E + 01 (5.04E − 02)	2.10E + 01 (5.68E − 02)	2.10E + 01 (4.28E − 02)	2.10E + 01 (5.58E − 02)	2.10E + 01 (4.53E − 02)
F9	0.00E + 00 (0.00E + 00)	0.00E + 00 (0.00E + 00)	0.00E + 00 (0.00E + 00)	0.00E + 00 (0.00E + 00)	**6.28E − 01 (3.44E + 00)**	0.00E + 00 (0.00E + 00)
F10	2.13E − 01 (9.69E − 02)	**2.29E − 01 (9.43E − 02)**	**2.26E − 01 (1.18E − 01)**	1.20E − 01 (7.26E − 02)	1.78E − 01 (9.01E − 02)	2.13E − 01 (1.07E − 01)
F11	0.00E + 00 (0.00E + 00)	0.00E + 00 (0.00E + 00)	0.00E + 00 (0.00E + 00)	**7.30E − 01 (1.50E + 00)**	**2.98E − 01 (1.63E + 00)**	0.00E + 00 (0.00E + 00)
F12	0.00E + 00 (0.00E + 00)	0.00E + 00 (0.00E + 00)	0.00E + 00 (0.00E + 00)	**4.79E + 01 (6.05E + 01)**	**4.52E + 01 (6.17E + 01)**	0.00E + 00 (0.00E + 00)
F13	0.00E + 00 (0.00E + 00)	0.00E + 00 (0.00E + 00)	0.00E + 00 (0.00E + 00)	**6.27E + 01 (7.44E + 01)**	**4.86E + 01 (7.05E + 01)**	0.00E + 00 (0.00E + 00)
F14	4.32E + 02 (1.84E + 02)	**4.40E + 02 (2.31E + 02)**	2.98E + 02 (1.33E + 02)	**6.70E + 02 (1.95E + 02)**	**6.38E + 02 (2.37E + 02)**	2.93E + 03 (1.59E + 02)
F15	6.34E + 03 (1.65E + 03)	**6.41E + 03 (1.16E + 03)**	**7.14E + 03 (2.99E + 02)**	3.59E + 03 (1.59E + 03)	3.44E + 03 (7.93E + 02)	**6.78E + 03 (2.38E + 02)**
F16	2.55E + 00 (2.90E − 01)	**2.63E + 00 (2.96E − 01)**	**2.61E + 00 (2.73E − 01)**	**2.60E + 00 (2.76E − 01)**	2.46E + 00 (4.30E − 01)	**2.68E + 00 (3.21E − 01)**
F17	1.70E + 01 (5.38E + 00)	**1.88E + 01 (7.44E + 00)**	1.22E + 01 (5.95E + 00)	**8.66E + 01 (4.49E + 01)**	**5.46E + 01 (2.34E + 01)**	**6.96E + 01 (6.97E + 00)**
F18	1.84E + 02 (1.72E + 01)	**1.91E + 02 (2.08E + 01)**	**1.86E + 02 (1.74E + 01)**	**2.01E + 02 (2.99E + 01)**	6.67E + 01 (2.11E + 01)	**1.89E + 02 (2.00E + 01)**
F19	4.96E + 00 (3.23E + 00)	**5.22E + 00 (4.28E + 00)**	**7.18E + 00 (5.48E + 00)**	**7.58E + 00 (2.83E + 00)**	**7.06E + 00 (5.19E + 00)**	**1.36E + 01 (3.22E + 00)**
F20	0.00E + 00 (0.00E + 00)	0.00E + 00 (0.00E + 00)	0.00E + 00 (0.00E + 00)	**2.94E + 00 (4.22E + 00)**	**2.05E + 00 (3.51E + 00)**	0.00E + 00 (0.00E + 00)
F21	4.00E + 02 (0.00E + 00)	4.00E + 02 (0.00E + 00)	4.00E + 02 (0.00E + 00)	4.00E + 02 (0.00E + 00)	4.00E + 02 (5.02E − 13)	4.00E + 02 (0.00E + 00)
F22	3.57E + 02 (1.63E + 02)	**4.45E + 02 (1.64E + 02)**	3.16E + 02 (1.72E + 02)	**7.35E + 02 (2.75E + 02)**	**8.76E + 02 (2.68E + 02)**	3.17E + 03 (2.76E + 02)
F23	6.48E + 03 (1.30E + 03)	**6.91E + 03 (4.48E + 02)**	**7.04E + 03 (3.51E + 02)**	3.54E + 03 (1.08E + 03)	3.76E + 03 (6.76E + 02)	**6.87E + 03 ()3.25E + 02**
F24	2.00E + 02 (4.62E − 02)	1.90E + 02 (3.06E + 01)	2.00E + 02 (2.65E − 02)	2.00E + 02 (9.58E − 02)	2.00E + 02 (7.21E − 02)	2.00E + 02 (2.88E − 02)
F25	2.22E + 02 (1.68E + 01)	2.22E + 02 (1.44E + 01)	2.20E + 02 (2.86E + 01)	**2.52E + 02 (3.76E + 01)**	**2.32E + 02 (2.73E + 01)**	2.20E + 02 (1.51E + 01)
F26	2.68E + 02 (6.13E + 01)	**2.93E + 02 (2.54E + 01)**	**2.93E + 02 (2.54E + 01)**	**2.99E + 02 (2.07E + 01)**	**2.93E + 02 (2.53E + 01)**	**2.87E + 02 (3.34E + 01)**
F27	1.61E + 03 (1.23E + 00)	1.61E + 03 (1.57E + 00)	1.62E + 03 (9.42E − 01)	1.66E + 03 (1.48E + 02)	1.61E + 03 (2.46E + 00)	1.61E + 03 (3.58E + 00)
F28	8.67E + 02 (1.54E + 01)	**8.74E + 02 (1.63E + 01)**	8.33E + 02 (1.39E + 02)	**1.03E + 03 (6.30E + 02)**	8.61E + 02 (2.09E + 02)	**9.65E + 02 (4.66E + 02)**
Count( − )	–	14	10	15	14	11
*Avg.Rank*	2.66	3.46	3.25	4.21	3.64	3.77
*Sort*	1	3	2	6	4	5

Note: The parameters that perform best on each function are bolded.

As can be seen from Table 3, the functions of the five improved algorithms performing perform IGTOA performance are 14, 10, 15, 14 and 11 respectively. This shows that the five improvements proposed in “Teacher assignment“, “Adaptive grouping”, “Improvement of update method for good group in student learning phase”, “Improvement of update method for average student group” , “Improvement of teacher teaching phase” Sections have some effectiveness. Furthermore, the rank of each algorithm obtained by Friedman detection is visible, with the smallest rank of the corresponding improvement algorithm after removing the good group improvement strategy of “Improvement of update method for good group in student learning phase” Section compared to IGTOA. This shows that among the five improvements, the improvement measure in “Improvement of update method for good group in student learning phase” Section has the greatest impact on the performance of the IGTOA algorithm, while several other improvement algorithms have little difference on the performance of IGTOA algorithm.

### Compared with other excellent algorithms

In order to fully investigate the performance of IGTOA algorithm, the algorithm, basic GTOA and four recently proposed representative optimization algorithms are analyzed in four aspects of convergence accuracy, convergence speed, stability and running time. Algorithms for comparison include IATTP(2021)^16^; MSMPSO(2018)^12^; ADN-RSN-PSO(2017)^11^; ESCA(2022)^17^. To ensure the fairness of the algorithm, the number of individuals in the population is *N* = 50, the test problem dimensions *D* = 30, 100, the maximum function evaluation times *MaxFEs* = 5000*D, and the remaining parameters are shown in Table 4.

**Table 4:** Initial parameters setting of each algorithm.

Algorithm	Initial parameters
IGTOA	*change_flag* = *30, Pg* = *0.1, l* = *10, N1* = *N2* = *0.2 N*
GTOA	*b* = *rand(0,1), c* = *1-b*
IATTP	*h1* = *h2* = *h3* = *0.5; h4* = *0.8; m* = *50, q* = *0.8*
MSMPSO	*pop1: c1* = *2.0, c2* = *1.0, c3* = *0.2; pop2: c1* = *0.1, c2* = *1.0, c3* = *2.0; pop3: c1* = *c2* = *c3* = *1.0; cycle* = *10*
ADN-RSN-PSO	*α*_*1*_ = *α*_*2*_ = *2.05, n*_*s*_ = *2, r*_*g*_ = *4, s*_*r*_ = *0.6, ρ* = *0.4, P*_*AND*_ = *0.2, L*_*0*_ = 0.35*range, L_*min*_ = *10*^–8^*range, x = *0.7298*
ESCA	*Pc* = *0.6, r2* = 2*pi*rand(0,1), r3 = 2*rand(0,1), r4 = *r5* = *r6* = *r7* = *r8* = *rand(0,1)*

#### Comparative experiment on algorithm convergence

Tables 5 and 6 respectively show the experimental results of each algorithm for solving the 30-and 100-dimensional function problems on the CEC2013 test set, with in-and out-parenthesis values being the standard deviation and mean of the optimal values obtained in 30 independent experiments. Among them, the bold data indicates that the corresponding algorithm has obtained the best solution effect on the test function, and the last line counts the number of functions for obtaining the best performance of each algorithm. To further compare the performance differences of the above algorithms, a Friedman test with a significant level of 0.05 was performed on the above data, and the specific results are shown in Table 7. Among them, the size of the p-value reflects the difference between the two algorithms in the current function. When the p-value is less than 0.05, it indicates that the IGTOA and the corresponding comparison algorithm have obvious differences in the current function, while otherwise, there is no significant difference between the two algorithms. In addition, the "+/=/−" in the last row indicates the number of functions that the IGTOA algorithm is significantly better than, not significantly different from, and significantly inferior to the corresponding comparison algorithm, respectively.

**Table 5:** Results of each algorithm on the 30-dimensional CEC2013 test set.

Function	IGTOA	GTOA	IATTP	MSMPSO	ADN-RSN -PSO	ESCA
F1	**0.00E + 00 (0.00E + 00)**	5.72E − 16 (3.13E − 15)	4.56E + 00 (1.69E + 00)	8.39E + 02 (6.62E + 02)	1.63E + 03 (5.39E + 03)	3.93E + 03 (9.25E + 02)
F2	**7.42E + 05 (2.91E + 05)**	3.32E + 06 (1.56E + 06)	1.23E + 07 (3.74E + 06)	2.44E + 07 (1.18E + 07)	2.48E + 07 (5.28E + 07)	1.71E + 08 (4.14E + 07)
F3	**0.00E + 00 (0.00E + 00)**	**0.00E + 00 (0.00E + 00)**	**0.00E + 00 (0.00E + 00)**	5.22E + 06 (2.86E + 07)	1.51E + 10 (8.25E + 10)	6.40E + 07 (2.89E + 08)
F4	4.79E + 03 (1.51E + 03)	8.89E + 03 (2.89E + 03)	**1.21E + 03 (7.82E + 02)**	1.08E + 04 (3.53E + 03)	7.36E + 04 (1.29E + 04)	5.51E + 04 (4.18E + 03)
F5	**0.00E + 00 (0.00E + 00)**	7.40E − 16 (1.85E − 15)	1.79E + 01 (1.03E + 01)	7.64E + 02 (5.20E + 02)	1.49E + 02 (5.99E + 02)	5.41E + 03 (1.29E + 03)
F6	**2.44E + 01 (2.47E + 01)**	4.47E + 01 (2.64E + 01)	8.49E + 01 (3.41E + 01)	2.26E + 02 (1.20E + 02)	7.31E + 01 (6.56E + 01)	5.33E + 02 (1.27E + 02)
F7	**0.00E + 00 (0.00E + 00)**	**0.00E + 00 (0.00E + 00)**	**0.00E + 00 (0.00E + 00)**	2.22E + 01 (4.43E + 01)	1.45E + 05 (4.45E + 05)	4.39E − 01 (2.40E + 00)
F8	**2.10E + 01 (4.81E − 02)**	**2.10E + 01 (5.67E − 02)**	2.11E + 01 (6.94E − 02)	**2.10E + 01 (4.02E − 02)**	2.14E + 01 (8.15E − 02)	**2.10E + 01 (5.61E − 02)**
F9	**0.00E + 00 (0.00E + 00)**	2.47E + 00 (7.58E + 00)	**0.00E + 00 (0.00E + 00)**	7.05E − 01 (3.07E + 00)	2.34E + 01 (1.49E + 01)	9.61E − 01 (2.98E + 00)
F10	**2.13E − 01 (9.69E − 02)**	5.25E + 00 (6.98E + 00)	2.74E + 01 (1.22E + 01)	2.40E + 02 (1.47E + 02)	3.58E + 01 (1.88E + 02)	1.06E + 03 (1.81E + 02)
F11	**0.00E + 00 (0.00E + 00)**	3.28E + 00 (5.80E + 00)	**0.00E + 00 (0.00E + 00)**	1.92E + 00 (3.46E + 00)	**0.00E + 00 (0.00E + 00)**	**0.00E + 00 (0.00E + 00)**
F12	**0.00E + 00 (0.00E + 00)**	6.99E + 01 (6.83E + 01)	**0.00E + 00 (0.00E + 00)**	1.21E + 02 (7.07E + 01)	3.14E + 02 (1.00E + 02)	2.46E + 01 (6.40E + 01)
F13	**0.00E + 00 (0.00E + 00)**	4.47E + 01 (7.02E + 01)	**0.00E + 00 (0.00E + 00)**	1.33E + 02 (7.77E + 01)	2.85E + 02 (6.21E + 01)	6.76E + 00 (3.70E + 01)
F14	**4.32E + 02 (1.84E + 02)**	2.76E + 03 (7.25E + 02)	7.61E + 03 (4.36E + 02)	4.01E + 03 (3.18E + 02)	9.03E + 02 (3.10E + 02)	7.42E + 03 (3.14E + 02)
F15	6.34E + 03 (1.65E + 03)	**4.86E + 03 (1.25E + 03)**	7.87E + 03 (6.44E + 02)	7.40E + 03 (3.83E + 02)	5.48E + 03 (1.63E + 03)	7.61E + 03 (3.15E + 02)
F16	**2.55E + 00 (2.90E − 01)**	2.56E + 00 (2.77E − 01)	3.79E + 00 (6.11E − 01)	2.60E + 00 (3.28E − 01)	2.77E + 00 (2.19E + 00)	2.80E + 00 (3.39E − 01)
F17	**1.70E + 01 (5.38E + 00)**	2.66E + 02 (1.01E + 02)	2.35E + 02 (2.10E + 01)	4.37E + 02 (1.17E + 02)	5.55E + 01 (4.96E + 01)	5.56E + 02 (6.00E + 01)
F18	**1.84E + 02 (1.72E + 01)**	2.43E + 02 (7.27E + 01)	2.56E + 02 (2.55E + 01)	5.17E + 02 (1.30E + 02)	1.01E + 03 (8.33E + 02)	5.48E + 02 (5.80E + 01)
F19	**4.96E + 00 (3.23E + 00)**	3.22E + 01 (2.59E + 01)	2.10E + 01 (1.59E + 00)	2.65E + 02 (4.83E + 02)	3.45E + 01 (2.03E + 01)	1.35E + 03 (1.12E + 03)
F20	**0.00E + 00 (0.00E + 00)**	8.60E + 00 (6.25E + 00)	6.46E − 01 (2.46E + 00)	1.31E + 01 (1.71E + 00)	1.47E + 01 (2.24E − 01)	2.50E + 00 (5.10E + 00)
F21	**4.00E + 02 (0.00E + 00)**	**4.00E + 02 (6.22E − 06)**	4.02E + 02 (4.41E − 01)	5.67E + 02 (1.06E + 02)	5.75E + 02 (6.18E + 02)	5.96E + 02 (3.92E + 01)
F22	**3.57E + 02 (1.63E + 02)**	3.05E + 03 (7.99E + 02)	8.10E + 03 (5.06E + 02)	4.54E + 03 (3.82E + 02)	1.76E + 03 (6.57E + 02)	7.60E + 03 (4.35E + 02)
F23	6.48E + 03 (1.30E + 03)	**4.57E + 03 (1.19E + 03)**	8.51E + 03 (4.30E + 02)	7.78E + 03 (3.86E + 02)	6.14E + 03 (1.35E + 03)	7.94E + 03 (4.14E + 02)
F24	**2.00E + 02 (4.62E − 02)**	2.39E + 02 (3.75E + 01)	2.00E + 02 (7.04E − 02)	2.19E + 02 (2.13E + 01)	2.58E + 02 (4.62E + 01)	3.02E + 02 (1.01E + 01)
F25	**2.22E + 02 (1.68E + 01)**	2.72E + 02 (2.16E + 01)	2.31E + 02 (2.66E + 01)	3.02E + 02 (2.04E + 01)	3.24E + 02 (4.51E + 01)	3.03E + 02 (3.18E + 00)
F26	2.68E + 02 (6.13E + 01)	3.15E + 02 (4.85E + 01)	2.49E + 02 (4.94E + 01)	**2.15E + 02 (3.42E + 01)**	3.70E + 02 (5.31E + 01)	3.79E + 02 (4.51E + 01)
F27	1.61E + 03 (1.23E + 00)	2.02E + 03 (3.61E + 02)	**3.40E + 02 (1.11E + 02)**	8.15E + 02 (2.08E + 02)	1.22E + 03 (1.83E + 02)	2.63E + 03 (3.28E + 01)
F28	**8.67E + 02 (1.54E + 01)**	2.02E + 03 (8.88E + 02)	9.96E + 02 (1.61E + 02)	2.54E + 03 (5.32E + 02)	3.30E + 03 (2.43E + 03)	2.31E + 03 (2.73E + 02)
	23	6	8	2	1	2

Note: The parameters that perform best on each function are bolded.

**Table 6:** Results of each algorithm on the 100-dimensional CEC2013 test set.

Function	IGTOA	GTOA	IATTP	MSMPSO	ADN-RSN -PSO	ESCA
F1	**3.18E − 29 (7.46E − 29)**	5.02E + 02 (1.13E + 03)	1.04E + 02 (1.95E + 01)	1.63E + 04 (6.68E + 03)	1.98E + 04 (6.10E + 04)	6.60E + 04 (7.29E + 03)
F2	**3.58E + 06 (6.13E + 05)**	4.68E + 07 (1.49E + 07)	1.02E + 08 (2.28E + 07)	3.78E + 08 (1.25E + 08)	5.12E + 07 (4.28E + 07)	9.46E + 08 (1.87E + 08)
F3	**0.00E + 00 (0.00E + 00)**	**0.00E + 00 (0.00E + 00)**	**0.00E + 00 (0.00E + 00)**	8.56E + 13 (1.25E + 14)	7.33E + 15 (4.01E + 16)	2.86E + 14 (5.01E + 14)
F4	1.32E + 04 (3.25E + 03)	3.80E + 04 (9.23E + 03)	**8.33E + 03 (1.64E + 03)**	1.02E + 05 (1.09E + 04)	2.65E + 05 (2.69E + 04)	2.07E + 05 (1.06E + 04)
F5	**3.75E − 25 (1.56E − 24)**	5.65E + 01 (1.10E + 02)	4.19E + 02 (8.29E + 01)	5.60E + 03 (3.06E + 03)	1.93E − 01 (8.39E − 01)	5.51E + 04 (1.16E + 04)
F6	**1.36E + 02 (4.83E + 01)**	4.40E + 02 (7.38E + 01)	5.56E + 02 (6.52E + 01)	2.41E + 03 (1.13E + 03)	2.52E + 02 (8.37E + 01)	7.91E + 03 (1.60E + 03)
F7	2.22E + 01(3.70E + 01)	3.62E + 03 (3.12E + 03)	**2.07E + 01 (4.42E + 01)**	1.33E + 04 (7.63E + 03)	4.33E + 07 (1.58E + 08)	8.77E + 03 (8.52E + 03)
F8	**2.13E + 01 (2.63E − 02)**	**2.13E + 01 (1.65E − 02)**	**2.13E + 01 (3.25E − 02)**	**2.13E + 01 (2.46E − 02)**	2.15E + 01 (3.21E − 02)	**2.13E + 01 (2.79E − 02)**
F9	**9.63E + 01 (5.33E + 00)**	1.13E + 02 (6.92E + 00)	1.13E + 02 (7.73E + 00)	1.27E + 02 (5.69E + 00)	1.37E + 02 (1.02E + 01)	1.26E + 02 (5.73E + 00)
F10	**1.36E − 01 (8.11E − 02)**	4.12E + 02 (1.56E + 02)	5.40E + 02 (1.01E + 02)	3.09E + 03 (1.17E + 03)	1.85E + 03 (6.28E + 03)	8.29E + 03 (1.11E + 03)
F11	1.62E + 01 (1.40E + 01)	2.63E + 02 (7.64E + 01)	7.11E − 01 (3.90E + 00)	1.46E + 02 (7.56E + 01)	**0.00E + 00 (0.00E + 00)**	**0.00E + 00 (0.00E + 00)**
F12	**3.77E + 02 (3.29E + 01)**	7.80E + 02 (1.07E + 02)	6.41E + 02 (7.48E + 01)	8.98E + 02 (9.77E + 01)	1.96E + 03 (6.52E + 02)	1.17E + 03 (1.27E + 02)
F13	**5.71E + 02 (6.85E + 01)**	1.04E + 03 (1.19E + 02)	6.53E + 02 (4.35E + 01)	1.10E + 03 (1.04E + 02)	2.32E + 03 (6.12E + 02)	1.18E + 03 (1.24E + 02)
F14	**3.96E + 03 (1.27E + 03)**	1.37E + 04 (1.43E + 03)	2.89E + 04 (3.99E + 03)	1.95E + 04 (1.09E + 03)	8.45E + 03 (2.97E + 03)	2.90E + 04 (5.48E + 02)
F15	2.88E + 04 (3.39E + 03)	**1.88E + 04 (4.96E + 03)**	3.01E + 04 (8.49E + 02)	3.04E + 04 (1.06E + 03)	1.90E + 04 (4.82E + 03)	2.92E + 04 (9.61E + 02)
F16	4.15E + 00 (2.46E − 01)	4.16E + 00 (2.31E − 01)	4.08E + 00 (2.75E − 01)	4.14E + 00 (2.38E − 01)	**3.19E + 00 (1.80E + 00)**	4.28E + 00 (2.50E − 01)
F17	**3.97E + 02 (1.64E + 02)**	2.87E + 03 4.40E + 02()	1.01E + 03 (3.89E + 01)	3.25E + 03 (5.78E + 02)	9.95E + 02 (2.30E + 03)	4.07E + 03 (3.43E + 02)
F18	**9.27E + 02 (5.57E + 01)**	2.47E + 03 (4.18E + 02)	1.01E + 03 (4.21E + 01)	3.39E + 03 (5.04E + 02)	5.64E + 03 (3.55E + 03)	4.06E + 03 (3.70E + 02)
F19	**5.75E + 01 (1.52E + 01)**	3.74E + 03 (2.06E + 03)	1.22E + 02 (1.76E + 01)	6.07E + 04 (7.04E + 04)	3.56E + 04 (1.94E + 05)	1.53E + 05 (4.83E + 04)
F20	**4.99E + 01 (1.46E − 01)**	**4.99E + 01 (1.60E − 01)**	5.00E + 01 (7.57E − 14)	5.00E + 01 (7.46E − 14)	5.00E + 01 (0.00E + 00)	5.00E + 01 (1.96E − 12)
F21	**4.00E + 02 (4.48E − 13)**	6.61E + 02 (2.53E + 02)	5.17E + 02 (2.42E + 01)	6.20E + 03 (9.25E + 02)	4.49E + 02 (3.34E + 02)	5.38E + 03 (4.38E + 02)
F22	**4.74E + 03 (1.27E + 03)**	1.56E + 04 (2.03E + 03)	3.05E + 04 (1.52E + 03)	2.12E + 04 (8.84E + 02)	9.03E + 03 (2.63E + 03)	2.99E + 04 (6.42E + 02)
F23	3.05E + 04 (8.50E + 02)	**2.23E + 04 (4.35E + 03)**	3.22E + 04 (7.02E + 02)	3.20E + 04 (1.52E + 03)	2.43E + 04 (2.89E + 03)	3.23E + 04 (6.62E + 02)
F24	**3.42E + 02 (5.81E + 01)**	5.74E + 02 (7.52E + 01)	3.65E + 02 (1.16E + 02)	5.63E + 02 (2.46E + 01)	1.03E + 03 (6.08E + 02)	6.08E + 02 (7.06E + 00)
F25	**5.29E + 02 (1.98E + 01)**	5.68E + 02 (2.84E + 01)	5.50E + 02 (2.29E + 01)	6.72E + 02 (3.26E + 01)	7.51E + 02 (1.07E + 02)	6.08E + 02 (5.25E + 00)
F26	**4.14E + 02 (6.88E + 01)**	5.92E + 02 (4.03E + 01)	4.47E + 02 (1.03E + 02)	5.55E + 02 (3.09E + 01)	6.74E + 02 (3.36E + 01)	6.99E + 02 (5.63E + 00)
F27	3.00E + 03 (6.89E + 02)	5.09E + 03 (2.62E + 02)	**1.88E + 03 (1.07E + 03)**	3.39E + 03 (3.50E + 02)	4.23E + 03 (4.98E + 02)	5.70E + 03 (5.59E + 01)
F28	9.02E + 03 (1.43E + 03)	1.33E + 04 (7.81E + 02)	**8.09E + 03 (3.06E + 03)**	1.49E + 04 (9.45E + 02)	2.29E + 04 (6.35E + 03)	1.41E + 04 (9.39E + 02)
−	20	5	6	1	2	2

Note: The parameters that perform best on each function are bolded.

**Table 7:** Results of the Wilcoxon rank sum test by IGTOA with other algorithms.

*p*-value (vs. IGTOA)										
Function	D = 30					D = 100				
	GTOA	IATTP	MSMPSO	ADN-RSN -PSO	ESCA	GTOA	IATTP	MSMPSO	ADN-RSN -PSO	ESCA
F1	0.0000 (−)	0.0000 (−)	0.0000 (−)	0.0000 (−)	0.0000 (−)	0.0000 (−)	0.0000 (−)	0.0000 (−)	0.0000 (−)	0.0000 (−)
F2	0.0000 (−)	0.0000 (−)	0.0000 (−)	0.0000 (−)	0.0000 (−)	0.0000 (−)	0.0000 (−)	0.0000 (−)	0.0000 (−)	0.0000 (−)
F3	1.0000 (=)	1.0000 (=)	0.3337 (−)	0.1608 (=)	0.0815 (=)	1.0000 (=)	1.0000 (=)	0.0000 (−)	0.0815 (=)	0.0000 (−)
F4	0.0000 (−)	0.0000 (+)	0.0000 (−)	0.0000 (−)	0.0000 (−)	0.0000 (−)	0.0000 (+)	0.0000 (−)	0.0000 (−)	0.0000 (−)
F5	0.0000 (−)	0.0000 (−)	0.0000 (−)	0.0000 (−)	0.0000 (−)	0.0000 (−)	0.0000 (−)	0.0000 (−)	0.0000 (−)	0.0000 (−)
F6	0.0000 −)	0.0000 (−)	0.0000 (−)	0.0000 (−)	0.0000 (−)	0.0000 (−)	0.0000 (−)	0.0000 (−)	0.0000 (−)	0.0000 (−)
F7	1.0000 (=)	1.0000 (=)	0.0003 (−)	0.0000 (−)	0.3337 (=)	0.0000 (−)	0.3953 (=)	0.0000 (−)	0.0000 (−)	0.0000 (−)
F8	0.4697 (=)	0.0000 (−)	0.0831 (=)	0.0000 (−)	0.0076 (−)	0.0030 (−)	0.1028 (=)	0.3353 (=)	0.0000 (−)	0.0030 (−)
F9	0.0815 (=)	1.0000 (=)	0.1608 (=)	0.0000 (−)	0.0815 (=)	0.0000 (−)	0.0000 (−)	0.0000 (−)	0.0000 (−)	0.0000 (−)
F10	0.0000 (−)	0.0000 (−)	0.0000 (−)	0.0000 (−)	0.0000 (−)	0.0000 (−)	0.0000 (−)	0.0000 (−)	0.0000 (−)	0.0000 (−)
F11	0.0001 (−)	1.0000 (=)	0.0001 (−)	1.0000 (=)	1.0000 (=)	0.0000 (−)	0.0000 (+)	0.0000 (−)	0.0000 (+)	0.0000 (+)
F12	0.0000 (−)	1.0000 (=)	0.0000 (−)	0.0000 (−)	0.0419 (−)	0.0000 (−)	0.0000 (−)	0.0000 (−)	0.0000 (−)	0.0000 (−)
F13	0.0014 (−)	1.0000 (=)	0.0000 (−)	0.0000 (−)	0.3337 (=)	0.0000 (−)	0.0000 (−)	0.0000 (−)	0.0000 (−)	0.0000 (−)
F14	0.0000 (−)	0.0000 (−)	0.0000 (−)	0.0000 (−)	0.0000 (−)	0.0000 (−)	0.0000 (−)	0.0000 (−)	0.0000 (−)	0.0000 (−)
F15	0.0000 (+)	0.0000 (−)	0.0000 (−)	0.0007 (+)	0.0000 (−)	0.0000 (+)	0.0009 (−)	0.0000 (−)	0.0000 (+)	0.5997 (=)
F16	0.9234 (=)	0.0000 (−)	0.5895 (=)	0.1297 (=)	0.0067 (−)	0.9705 (=)	0.3255 (=)	0.8187 (=)	0.0001 (+)	0.0905 (=)
F17	0.0000 (−)	0.0000 (−)	0.0000 (−)	0.0000 (−)	0.0000 (−)	0.0000 (−)	0.0000 (−)	0.0000 (−)	0.3711 (=)	0.0000 (−)
F18	0.0012 (−)	0.0000 (−)	0.0000 (−)	0.0000 (−)	0.0000 (−)	0.0000 (−)	0.0000 (−)	0.0000 (−)	0.0000 (−)	0.0000 (−)
F19	0.0000 (−)	0.0000 (−)	0.0000 (−)	0.0000 (−)	0.0000 (−)	0.0000 (−)	0.0000 (−)	0.0000 (−)	0.0000 (−)	0.0000 (−)
F20	0.0000 (−)	0.1608 (=)	0.0000 (−)	0.0000 (−)	0.0110 (−)	0.4113 (=)	0.0419 (−)	0.3414 (=)	0.0419 (−)	0.0419 (−)
F21	1.0000 (=)	0.0000 (−)	0.0000 (−)	0.0000 (−)	0.0000 (−)	0.0000 (−)	0.0000 (−)	0.0000 (−)	0.0002 (−)	0.0000 (−)
F22	0.0000 (−)	0.0000 (−)	0.0000 (−)	0.0000 (−)	0.0000 (−)	0.0000 (−)	0.0000 (−)	0.0000 (−)	0.0000 (−)	0.0000 (−)
F23	0.0000 (+)	0.0000 (−)	0.0000 (−)	0.0012 (+)	0.0000 (−)	0.0000 (+)	0.0000 (−)	0.0001 (−)	0.0000 (+)	0.0000 (−)
F24	0.6607 (=)	0.0000 (−)	0.0000 (−)	0.0024 (−)	0.0000 (−)	0.0000 (−)	0.1154 (=)	0.0000 (−)	0.0000 (−)	0.0000 (−)
F25	0.0000 (−)	0.5106 (=)	0.0000 (−)	0.0000 (−)	0.0000 (−)	0.0000 (−)	0.0013 (−)	0.0000 (−)	0.0000 (−)	0.0000 (−)
F26	0.9821 (=)	0.5591 (=)	0.0303 (+)	0.0000 (−)	0.0000 (−)	0.0000 (−)	0.0046 (−)	0.0000 (−)	0.0000 (−)	0.0000 (−)
F27	0.1840 (=)	0.0000 (+)	0.0000 (+)	0.0000 (+)	0.0000 (−)	0.0000 (−)	0.0000 (+)	0.0377 (−)	0.0000 (−)	0.0000 (−)
F28	0.0000 (−)	0.0000 (−)	0.0000 (−)	0.0000 (−)	0.0000 (−)	0.0000 (−)	0.7172 (=)	0.0000 (−)	0.0000 (−)	0.0000 (−)
**+/=/−**	2/9/17	2/9/17	2/3/23	3/3/22	0/5/23	2/3/23	3/6/19	0/3/25	4/2/22	1/2/25

As can be seen from the data in Tables 5, 6 and 7, when the dimension of the test function is 30, compared with the IGTOA algorithm, the basic GTOA only achieved significantly better results on F15 and F23, in addition to 9 functions, including F3, F7, F8, F9, F16, F21, F24, F26 and F27, while achieving significantly worse results on the remaining 17 functions; the IATTP algorithm only achieved significantly better results on F4 and F27, in addition to comparable results on 9 functions, including F3, F7, F9, F11, F12, F13, F20, F25 and F26, and achieved significantly worse results on the remaining 17 functions; the MSMPSO algorithm has achieved significantly better results only on F26 and F27, in addition to comparable results on 3 functions, including F8, F9 and F16, while achieving significantly worse results on the remaining 23 functions; the ADN-RSN-PSO algorithm has achieved significantly better results only on F15, F23 and F27, except in F3, F11, F 11 and F16, but in the remaining 22 functions; and the ESCA algorithm showed similar performance on only 5 functions, including F3, F7, F9, F11 and F13, but performed significantly worse on the remaining 23 functions. When the dimension of the test function is 100, the basic GTOA shows significantly better performance on only 2 functions compared with the IGTOA algorithm, but shows worse performance on all 23 functions; the IATTP algorithm was significantly better on only 3 functions but worse on 19 functions; the MSMPSO algorithm was not significantly better on any function but significantly worse on 25 functions; the ADN-RSN-PSO algorithm was significantly better on 4 but worse on 22 functions and the ESCA was significantly worse on 25 functions. In Conclusion Section, we show that IGTOA has obvious advantages in convergence accuracy over the remaining five algorithms. In addition, as the dimension of the optimization problem increases, the advantages of the IGTOA algorithm are also greater. To further compare the comprehensive performance of each algorithm on all functions, Table 8 presents the results of Friedman detection.

**Table 8:** The Friedman detection results for each algorithm.

		IGTOA	GTOA	IATTP	MSMPSO	ADN-RSN -PSO	ESCA
D = 30	*Avg.Rank*	1.63	2.93	3.02	4.05	4.48	4.89
	*Sort*	1	2	3	4	5	6
D = 100	*Avg.Rank*	1.61	3.07	2.82	4.38	4.07	5.04
	*Sort*	1	3	2	5	4	6

As can be seen from the Friedman test results in Table 8, the rank of IGTOA is significantly lower than the other five methods, indicating that the IGTOA algorithm performs best in terms of convergence accuracy.For 30-dimensional function optimization problems, the comprehensive performance of each algorithm is IGTOA > GTOA > IATTP > ADN-RSN-PSO > MSMPSO > ESCA; for 100-dimensional function optimization problems, the comprehensive performance of each algorithm is IGTOA > IATTP > GTOA > ADN-RSN-PSO > MSMPSO > ESCA.

#### Comparative test of the convergence rate of the algorithm

**Figure 8:**
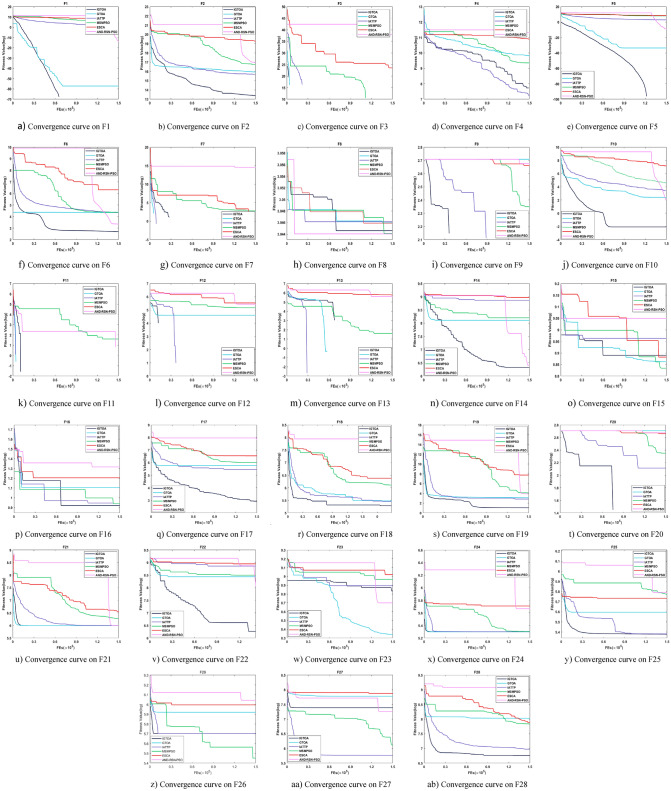
Convergence curves of each algorithm on the test suit.

In order to compare the convergence rate of the algorithm more intuitively, Fig. 8 gives the iterative process curve where each algorithm is run randomly once when the test function dimension is 30.The horizontal and vertical coordinates represent the logarithm of the function evaluation times and the fitness values, respectively.Parameter settings for each algorithm are performed as in Table 4.

As can be seen in Fig. 8. For the functions F1, F3, F5, F7, F9, F11, F12, F13, and F20, the IGTOA all converge to the theoretical optimal results; the ESCA converges to the theoretical optimum on F3, F9, F11, and F13; the IATTP algorithm converges to the theoretical optimum on F3, F7, F9, F11, F12, and F13; the GTOA converges to the theoretical optimum on the F3, F7, F9, F11, and F13; the ADN-RSN-PSO converges to the theoretical optimum only on the F11; while MSMPSO does not obtain the theoretical optimal results on any function. Compared with IGTOA, GTOA and IATTP showed faster convergence on F9, F11 and F13, while ESCA converged only faster on F11, while other algorithms converged slower on the remaining functions, including F1, F3, F5, F7, F12 and F20. For the remaining 19 functions, each algorithm converged to the local optima, including F2, F4, F6, F8, F10, F14-F19 and F21-F28. For F2, F14, F19 and F21, IGTOA only converged slightly slower than GTOA in the early evolution, but all faster than the other four algorithms, especially in the late evolution, IGTOA showed faster convergence than the other five algorithms. For F4, IGTOA converges slower than IATTP, but faster than the other 4 contrast algorithms. For F15, F23 and F26, the IGTOA showed the fastest convergence compared with the other five algorithms, whereas the IGTOA decreased.Later in evolution, IGTOA converged only faster on F15 than IATTP, slower on F23 than GTOA and ADN-RSN-PSO, and only slightly slower than MSMPSO on F26. For F16, IGTOA converges only converged slightly slower than MSMPSO in early evolution, but by later evolution, IGTOA showed the fastest convergence. For F24, IGTOA showed the fastest convergence rate in the early evolution, slowing down as evolution progressed and being comparable to that of IATTP and GTOA. For F27, IGTOA converged slightly slower than MSMPSO and IATTP in the early evolution, the convergence of each algorithm decreased, but ADN-RSN-PSO decreased more slowly, and by the later evolution ADN-RSN-PSO, MSMPSO and IATTP all converged faster than IGTOA. But for other functions, including F6, F10, F17, F18, F22, F25, and F28, IGTOA showed the fastest convergence rate compared to the other five evolutionary algorithms. In Conclusion Section, IGTOA has some advantages in convergence speed over the other five algorithms.

#### Comparative test of the algorithm stability

**Figure 9:**
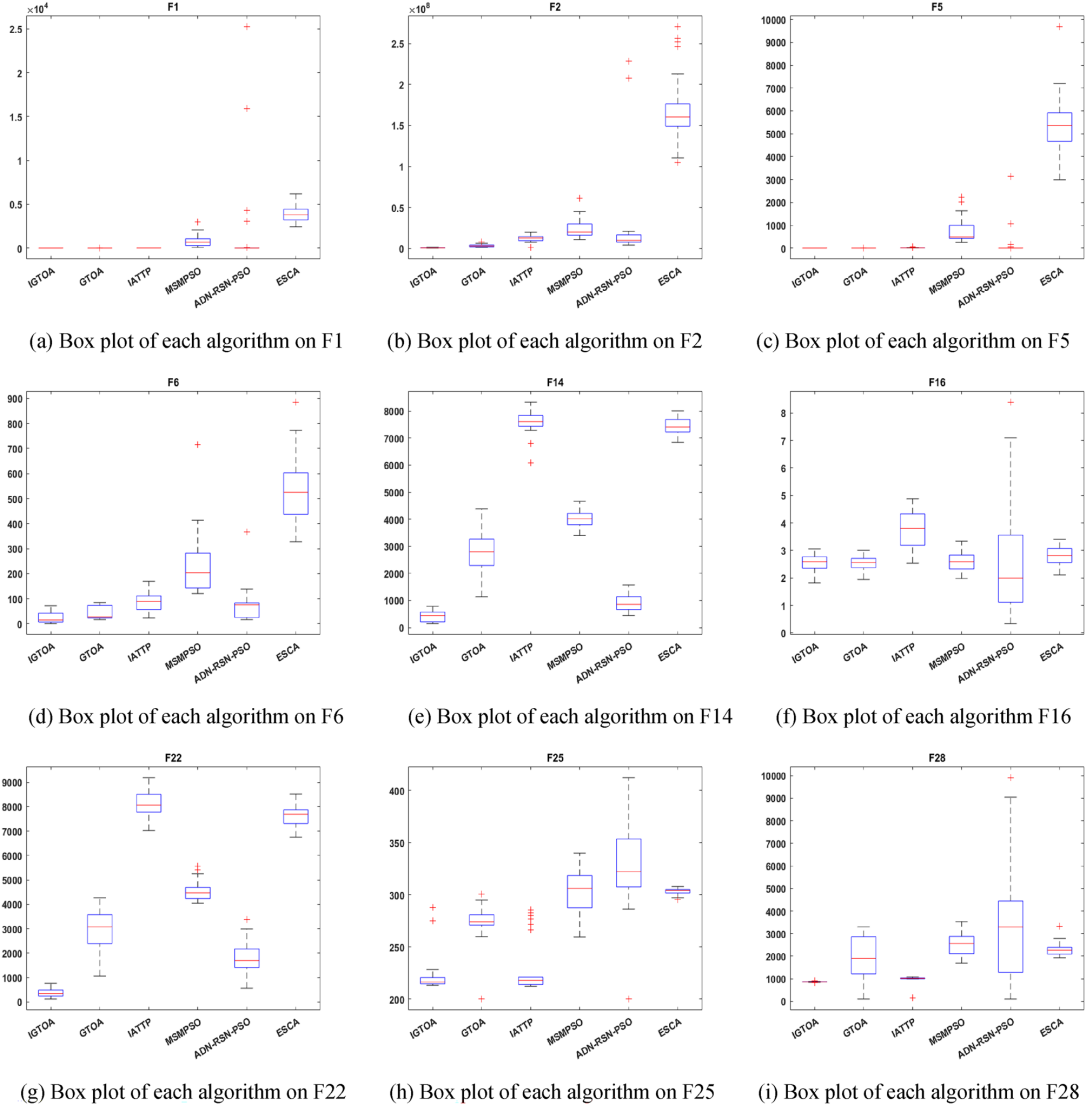
Box plots of the 6 algorithms on the test function.

To intuitively compare the stability of each algorithm, we draw the box plot of the optimal results obtained from 30 independent runs of each algorithm.Limited to space, this section selects only nine different types of functions for comparison, including: F1, F2 and F5 in uni-modal functions; F6, F14 and F16 in multi-modal functions; and F22, F25 and F28 in combined functions. As shown in Fig. 9.

Figure 9 follows from the fact that for the uni-modal functions F2 and F5 and the multi-modal functions F22 and F14, IGTOA is more stable than the other five algorithms.For the uni-modal function F1, the stability of IGTOA and IATTP was flat, and both significantly outperformed the other four algorithms, including GTOA, MSMPSO, ADN-RSN-PSO, and ESCA.For multi-modal function F16, IGTOA is only slightly less stable than GTOA; for combined function F25, IGTOA is slightly less stable than ESCA, but its solution accuracy is significantly better than ESCA. In Conclusion Section, the IGTOA algorithm has certain stability advantages compared to the other five algorithms.

#### The algorithm running time comparison test

To further compare the complexity of each algorithm, this section counts the average running time of IGTOA and other comparison algorithms running independently for 30 times under the same number of function evaluation times. The specific results are shown in Table 9. The dimension of the test function is 30, the maximum number of evaluation is *Max_FEs* = 5000*D, and the other parameter settings are shown in Table 4.

**Table 9:** Comparison of time complexity between IGTOA and other algorithms.

Mean-times/s						
Function	IGTOA	GTOA	IATTP	MSMPSO	ADN-RSN -PSO	ESCA
F1	5.19	1.04	4.29	1.49	0.89	1.40
F2	6.81	2.34	5.32	2.73	2.16	2.64
F3	6.12	1.69	5.27	2.17	1.67	2.16
F4	6.26	1.93	4.92	2.30	1.73	2.21
F5	5.58	1.39	4.35	1.89	1.35	1.81
F6	5.99	1.38	4.24	1.80	1.23	1.72
F7	8.34	3.68	7.13	4.56	4.08	4.65
F8	7.64	3.45	6.26	3.71	3.15	4.65
F9	32.58	28.65	31.71	28.95	28.40	29.51
F10	6.65	2.27	5.33	2.83	2.20	2.57
F11	6.54	2.26	5.24	2.84	2.4121	2.84
F12	8.14	3.39	6.69	4.15	3.46	3.91
F13	8.96	4.72	7.96	5.05	4.60	4.80
F14	6.35	2.01	5.1 9	2.41	1.87	2.41
F15	7.13	2.50	5.54	2.92	2.30	2.82
F16	24.17	19.96	23.11	20.42	19.91	20.29
F17	6.45	1.84	4.33	2.26	1.72	2.08
F18	7.08	2.44	5.11	2.82	2.20	2.62
F19	6.16	1.59	4.02	2.06	1.44	1.88
F20	6.66	2.47	5.28	2.99	2.43	3.19
F21	10.91	6.88	9.40	6.72	6.55	6.67
F22	10.20	6.27	9.39	6.79	6.13	6.58
F23	11.92	7.43	9.70	7.10	6.51	7.32
F24	39.87	35.13	38.11	35.55	34.95	36.23
F25	39.70	35.16	38.08	35.44	34.97	35.73
F26	42.73	38.45	41.52	38.70	38.20	38.73
F27	41.88	37.60	40.04	37.60	37.15	37.73
F28	15.41	11.05	13.82	11.94	11.22	10.61

As seen from Table 9, for the unimodal function F1-F20, I GTOA runs for slightly longer times compared to GTOA, MSMPSO, ADN-RSN-PSO, and ESCA. For the combined functions F21-F28, the running time of each algorithm is not very different. However, the running time of the algorithms is not much different, which means that the time complexity of I GTOA is slightly higher compared with G T O A and other contrast algorithms. This is due to the multiple improvement strategies employed by IGTOA, requiring more manipulation when looking for better individuals. Combined with the convergence rate, with the same convergence accuracy, IGTOA does not increase compared with the other algorithms.

### Comparison of the engineering application effect

In order to further compare the effects of IGTOA algorithm and other comparison algorithms in practical application, this section uses each algorithm to handle the cooperative beam forming optimization problem. The cooperative beam forming optimization problem is a typical problem in the antenna array. By optimizing the amplitude and phase of the emission signal weight of each cooperative node, the peak side valve level PSL minimization as shown in formula (17) is realized.

17 $$PSL = 20\log_{10} \frac{{\max \left| {AF(\theta_{SL} ,w)} \right|}}{AF(\phi ,w)}$$

where, ${\text{AF}}\left( {\theta ,w} \right)$ represents the array factor, as shown in formula (18). *φ* is the main beam direction. $\theta_{SL}$ is the direction corresponding to the peak point in the range $\theta \in \left[ { - \pi ,\phi } \right) \cup \left( {\phi ,\pi } \right]$ beam chart except for the main lobe peak point, is called the lateral lobe direction. The denominator ${\text{AF}}\left( {\phi {,}w} \right)$ is the main beam power and the molecule $\max \left| {{\text{AF}}\left( {\theta_{SL} ,w} \right)} \right|$ is the maximum beam power in the side flap.

18 $${\text{AF}}\left( {\theta ,w} \right) = \sum\limits_{k = 1}^{k} {w_{k} e^{{j\left( {2\pi /\lambda } \right)r_{k} \left[ {\cos \left( {\theta - \psi_{k} } \right)} \right]}} }$$

where, *w*_*k*_ is the complex number weight coefficient of the signal emitted by the *k*-th cooperative node, as shown in formula (19).

19 $$w_{k} = \xi_{k} e^{{j\alpha_{k} }}$$

where, $\xi_{k}$ and $\alpha_{k}$ are the amplitude and initial phase of the emission signal weights of the *k*-th cooperative node, respectively, and $\xi \in \left[ {0,1} \right]$, $\alpha \in \left[ { - \pi {\kern 1pt} ,\pi } \right]$.

**Figure 10:**
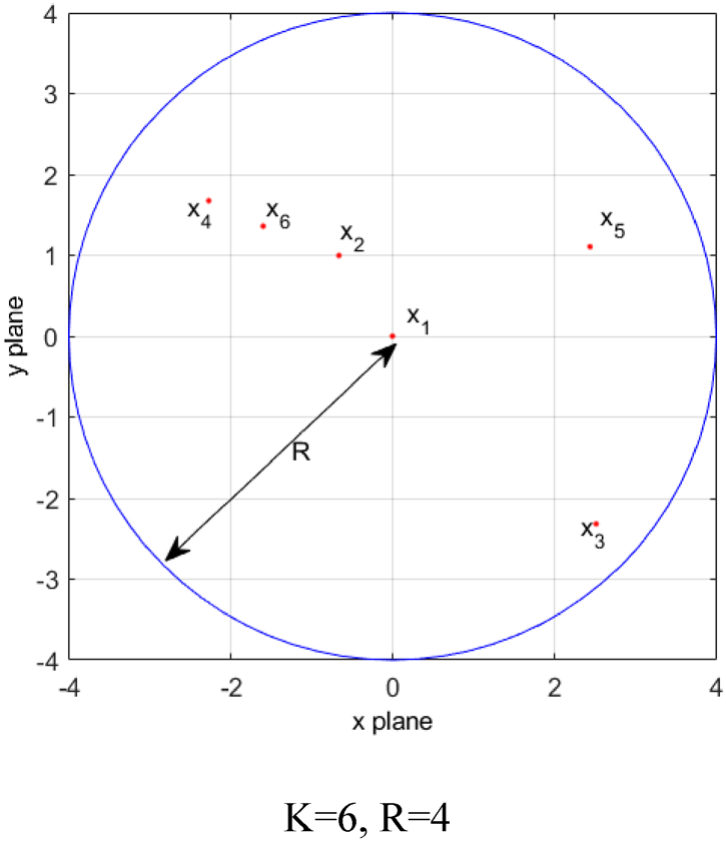
Distribution of the cooperative nodes.

**Figure 11:**
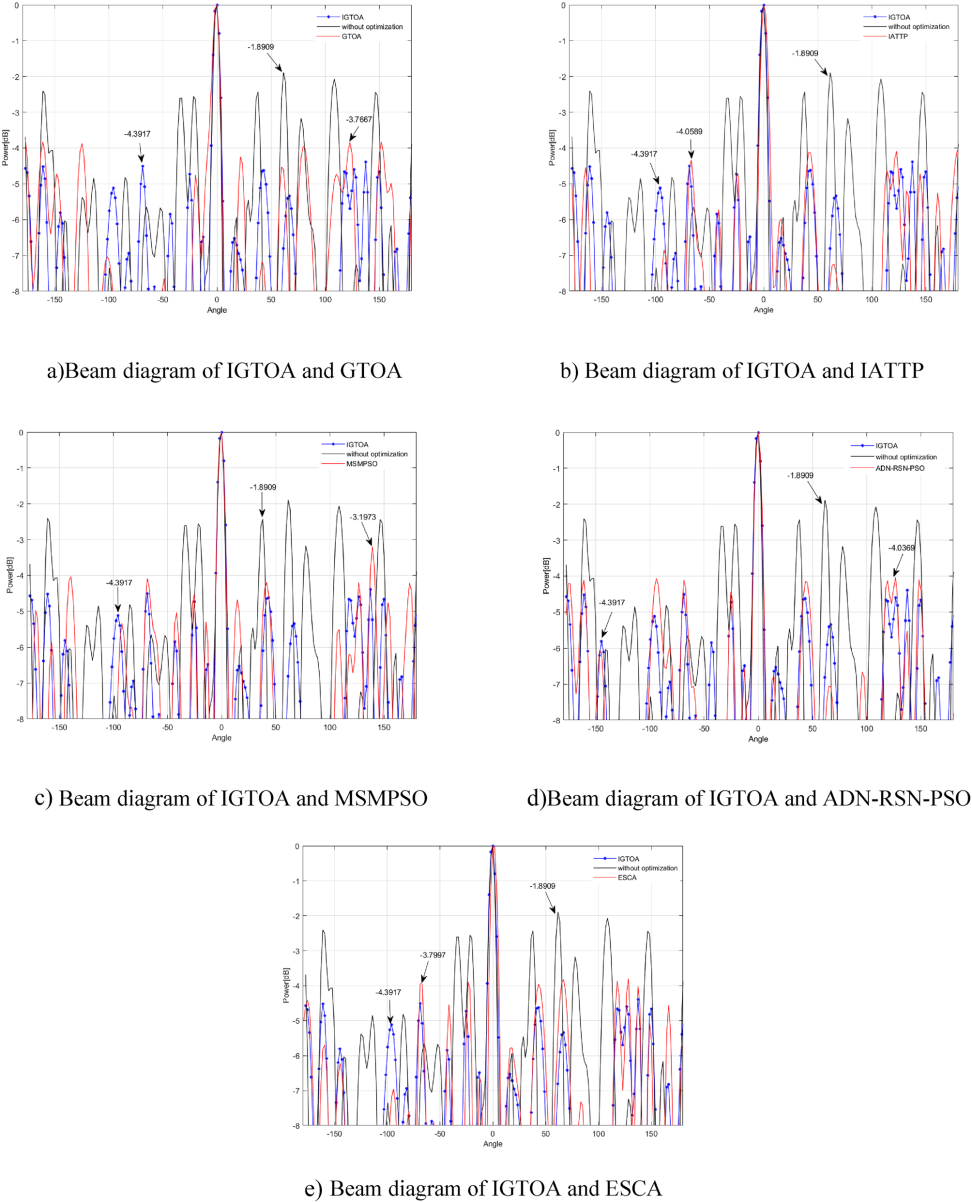
Beam map of IGTOA and each contrast algorithm in a rectangular coordinate frame.

The beam forming scenario in this section is shown in Fig. 10. Among them, the wavelength of the sending signal is $\lambda$, and the six cooperative nodes are distributed in the circle domain with a radius of $4\lambda$, and one cooperative node is located in the center of the circle domain. Each algorithm is optimized as the objective function shown in formula (17). For comparative fairness, in this experiment, the problem scale *N* is 50, the maximum function evaluation times *Max_FEs* = 5000*D, the number of nodes *k* = 6, the polar radius *r*_*k*_ = 4, and other parameters are shown in Table 4. In order to avoid the adverse effects of contingency on the algorithm evaluation, each algorithm runs independently for 10 times, and selects the best collaborative beam optimization scheme corresponding to the PSL median of each algorithm is compared. Figure 11 intuitively gives the beam diagram of IGTOA and each comparison algorithm in the right Angle coordinate system, and then the PSL corresponding to each algorithm is annotated in the graph.

As can be seen from Fig. 11, for the above collaborative beam forming scenario, the best PSL obtained from GTOA, IATTP, MSMPSO, ADN-RSN-PSO, ESCA, and IGTOA are: − 3.7667 dB, − 4.0599 dB, − 3.1973 dB, − 4.0369 dB, − 3.7997 dB and − 4.3917 dB, respectively. Each algorithm achieved better cooperative beam optimization than unoptimized (− 1.8909 dB), and IGTOA achieved the best synergistic beam optimization than the other five algorithms. In Conclusion Section, the proposed IGTOA also has excellent performance in engineering applications.

## Conclusion

This paper proposes an improved algorithm-IGTOA, which assigns teachers by probability and introduces different excellent genes in the group to ensure the population diversity; at the same time, the adaptive grouping method, combined with the different learning abilities of students in the two groups, put forward suitable search methods and learning methods, balancing the diversity loss rate in the evolution process and the algorithm convergence rate; in addition, this paper proposes a population reconstruction mechanism that starts with whether the population optimal individual has continuous changes and provides new genes for the population while maintaining excellent genes, which ensures the convergence rate of the algorithm and better maintains the population diversity; finally, simulation results from multiple experiments of this algorithm in the CEC2013 test suite show that IGTOA has good comprehensive performance, and IGTOA has obvious advantages in convergence speed and solution accuracy compared with many other comparative algorithms.

## Data Availability

The datasets used or analysed during the current study available from the corresponding author on reasonable request.
